# Detection and removal of barcode swapping in single-cell RNA-seq data

**DOI:** 10.1038/s41467-018-05083-x

**Published:** 2018-07-10

**Authors:** Jonathan A. Griffiths, Arianne C. Richard, Karsten Bach, Aaron T. L. Lun, John C. Marioni

**Affiliations:** 1 Cancer Research UK Cambridge Institute, University of Cambridge, Cambridge, CB2 0RE United Kingdom; 2 Cambridge Institute for Medical Research, University of Cambridge, Cambridge, CB2 0XY United Kingdom; 3 Department of Pharmacology, University of Cambridge, Cambridge, CB2 1PD United Kingdom; 4 European Molecular Biology Laboratory, European Bioinformatics Institute (EMBL-EBI), Wellcome Genome Campus, Hinxton, CB10 1SD United Kingdom; 5 Wellcome Sanger Institute, Wellcome Genome Campus, Hinxton, CB10 1SA United Kingdom

## Abstract

Barcode swapping results in the mislabelling of sequencing reads between multiplexed samples on patterned flow-cell Illumina sequencing machines. This may compromise the validity of numerous genomic assays; however, the severity and consequences of barcode swapping remain poorly understood. We have used two statistical approaches to robustly quantify the fraction of swapped reads in two plate-based single-cell RNA-sequencing datasets. We found that approximately 2.5% of reads were mislabelled between samples on the HiSeq 4000, which is lower than previous reports. We observed no correlation between the swapped fraction of reads and the concentration of free barcode across plates. Furthermore, we have demonstrated that barcode swapping may generate complex but artefactual cell libraries in droplet-based single-cell RNA-sequencing studies. To eliminate these artefacts, we have developed an algorithm to exclude individual molecules that have swapped between samples in 10x Genomics experiments, allowing the continued use of cutting-edge sequencing machines for these assays.

## Abstract (web summary)

DNA barcode swapping results in mislabelling of sequencing reads between multiplexed samples. Here, the authors investigate the severity and consequences of barcode swapping for single-cell RNA-seq data, and develop a computational method to exclude swapped reads.

## Introduction

**Fig. 1:**
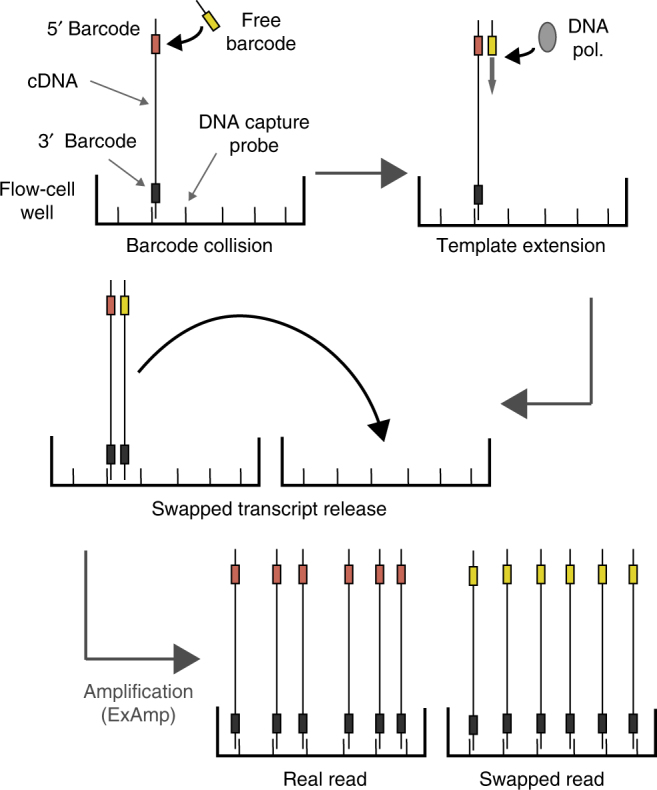
A schematic of the mechanism for barcode swapping, as proposed by Sinha et al.^1^. On new models of the Illumina sequencing machines, flow cell seeding and DNA amplification take place simultaneously, without any washes of the flow cell between steps. As a result, free sample indexing barcodes remain in solution and can be inadvertently extended using DNA molecules from libraries with different barcodes as templates. The transfer of mislabelled molecules between nanowells of the flow cell results in clustering and sequencing of incorrectly labelled DNA molecules

Recent reports have shown that the DNA barcodes used to label multiplexed libraries can “swap” on patterned flow-cell Illumina sequencing machines, including the HiSeq 4000, HiSeq X, and NovaSeq^1,2^. This results in mislabelling whereby reads assigned to one sample are derived from molecules in another, thus compromising the interpretation of many 'omics assays (Fig. 1). Barcode swapping is particularly problematic for single-cell RNA sequencing (scRNA-seq) experiments, where many libraries are routinely multiplexed together. For example, barcode swapping could lead to cells that appear to falsely express particular marker genes, or yield spurious correlation patterns that may confound clustering and other analyses.

The severity and consequences of barcode swapping in scRNA-seq studies remain poorly understood. Sinha et al.^1^ estimated swapping rates of “up to 5–10%” from a plate-based scRNA-seq experiment; however, these estimates were obtained from only two wells in a single micro-well plate. The lack of replication makes it difficult to generalize the results to other scRNA-seq studies. Furthermore, the effect of barcode swapping on high-throughput droplet-based scRNA-seq protocols^3^ has not been explored. This is a key consideration due to the increasing use of droplet-based methods for large-scale single-cell studies^4,5^ where many samples are necessarily multiplexed together for efficient sequencing.

Here, we robustly quantify the fraction of swapped reads in each of two plate-based single-cell RNA sequencing datasets. We found that approximately 2.5% of reads were mislabelled between samples on HiSeq 4000, and observed no correlation between the swapped fraction of reads and the concentration of free barcode across plates. Furthermore, we demonstrate that barcode swapping can generate complex but artefactual cell libraries in droplet-based scRNA-seq data. To eliminate these artefacts, we developed a computational method to exclude swapped reads in 10x Genomics experiments, enabling the continued use of cutting-edge sequencing machines for droplet-based assays.

## Results

### Barcode swapping in plate scRNA-seq experiments

A number of widely-used scRNA-seq library preparation methods isolate and process individual cells in wells of a microwell plate before performing library preparation in parallel^6–8^. A unique combination of sample barcodes characterises the library associated with each cell, usually by adding a different barcode to each end of a cDNA molecule. One barcode typically indexes the row position for each cell on the microwell plate, while the other barcode indexes the column position. Swapping of either or both barcodes therefore moves reads between cell libraries. We used two independent plate-based scRNA-seq datasets to quantify the swapping fraction, i.e., the fraction of all cDNA reads across all sequencing libraries multiplexed on a single flow-cell lane that were mislabelled.

**Fig. 2:**
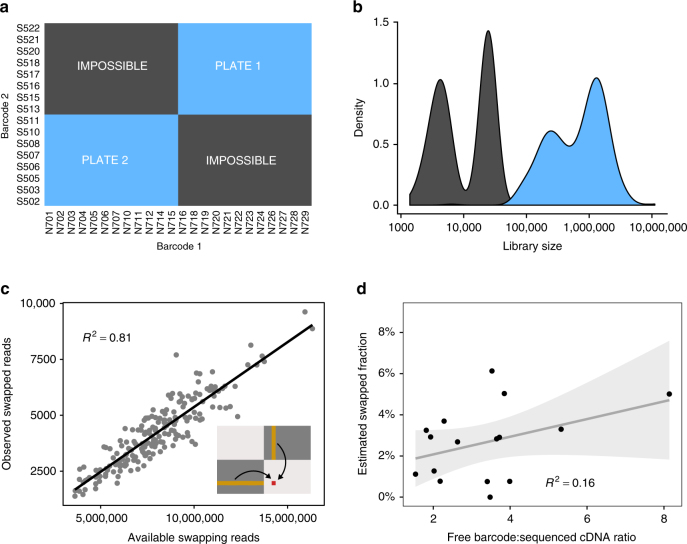
Characterization of barcode swapping in plate-based scRNA-seq experiments. **a** The experimental design of the Richard dataset. Two 96-well plates of cells were multiplexed for sequencing. Expected barcode combinations are marked in blue, while impossible barcode combinations are marked in grey. **b** Distribution of the library sizes (i.e., number of mapped reads) in the expected and impossible barcode combinations. **c** Library size of each impossible combination (observed swapped reads), plotted against the sum of the library sizes of the expected combinations that share exactly one barcode with that impossible combination (available swapping reads). An example is illustrated graphically in the inset Figure for one impossible combination (red) and the contributing expected combinations (orange). The gradient represents the fraction of available reads from the expected combinations that swap into each impossible combination. **d** Estimated swapping fractions for different plates of the Nestorowa et al. [10] dataset, plotted against the ratio of the concentration of free barcode to the concentration of cDNA of the correct length for sequencing. A linear regression fit is shown with its 95% confidence interval. The slope of the fitted line is not significantly different from 0 (*p* = 0.129)

In the first dataset (Richard et al.^9^, see Methods and Supplementary Note 3), two plates of single mouse T cells were multiplexed for sequencing on a HiSeq 4000 instrument. Each of these plates used entirely different sets of column and row barcodes: none of the barcodes were reused between the plates (Fig. 2a). As such, there exists a set of barcode combinations that should contain zero reads (“impossible” combinations), as the two sets of barcodes for these combinations were never mixed during the experiment. However, reads mappable to the mouse genome were still present in the impossible combinations at approximately 1% of the frequency in the expected combinations (Fig. 2b). This cannot be explained by contamination from free-floating nucleic acids, which can only affect the expected combinations used during library preparation. Indeed, the number of reads in each impossible combination was proportional to the number of reads in the real cell libraries that shared exactly one barcode with the impossible combination (Fig. 2c). This is consistent with read misassignment to an impossible combination due to swapping of a single barcode from the pool of cDNA in the expected combinations.

To estimate the swapped fraction in the Richard et al. dataset^9^, we regressed the library size of the impossible combinations against the summed library sizes of the real cells that shared exactly one barcode (Fig. 2c, see Methods and Supplementary Note 3 for more details). This yielded an estimate of the swapped read fraction of 2.18 ± 0.08%. For comparison, we repeated this procedure on the same libraries sequenced on HiSeq 2500, yielding a much lower swapped fraction estimate of 0.22 ± 0.01%. This is consistent with the proposed mechanism of barcode swapping on the new Illumina machines. Notably, our estimates are calculated over many wells with impossible combinations, offer an estimate of uncertainty, and are robust to contamination. This represents an improvement over previous estimates^1^, which only sought to technically demonstrate the existence of swapping by considering two wells of a single plate.

In the second dataset (Nestorowa et al.^10^, see Methods and Supplementary Note 4), we considered plates of single cells whose libraries had been sequenced on both the HiSeq 2500 and 4000. We modelled each cell’s gene expression profile in the HiSeq 4000 data as a linear combination of contributions from the HiSeq 2500 data. Specifically, for each cell, we considered contributions from itself, cells that share exactly one barcode, and cells that share no barcodes (see Methods and Supplementary Note 4 for details). The relative contribution from other cells was used to estimate the swapping fraction across all cells in the plate. Across 16 independent plates of single cells (each of which was sequenced separately), we estimated a range of swapped read fractions with mean 2.275 ± 0.359%, consistent with the first experiment (Fig. 2d). We also observed that nearly all expressed genes were affected by swapping (Methods and Supplementary Note 4), consistent with its global effects on the pool of sequencable DNA.

Given the range of estimated swapping fractions in the Nestorowa et al. data^10^, we reasoned that we could identify the factors driving barcode swapping by considering the library characteristics of each plate. Specifically, we investigated the association with the amount of free library barcode, which had previously been linked to swapping rates^1^. We used Agilent’s Bioanalyzer Expert 2100 software to quantify the amount of free barcode (DNA lengths 45–70 bp) and the amount of sequencable cDNA (400-800 bp) in the multiplexed library from each plate. However, we did not observe a strong correlation of swapping fraction estimates with the ratio of free barcode to sequencable cDNA (Fig. 2d). Similarly, no correlation was observed with the total amount of free barcode per plate, or the ratio of free barcode concentration to mappable reads (Methods and Supplementary Note 4). This suggests that the extent of barcode swapping is not primarily determined by the amount of free barcode in experiments using typical barcode concentrations.

### Removing effects of barcode swapping in plate experiments

The most obvious solution for barcode swapping is to use a sequencing machine that does not use a patterned flow-cell, which we have shown reduces the swapping rate by an order of magnitude. Where this is not possible, an approach for computationally “unmixing” the expression profiles has been described^11^, where the swapping rate is estimated using the subset of genes that are detected above a certain threshold in only a single cell on a plate.

For general plate-based scRNA-seq experiments, we recommend the use of an experimental design similar to that in the Richard et al. dataset^9^ (Fig. 2a). By leaving a fraction of possible barcode combinations unoccupied, a researcher can robustly estimate the swapped fraction of reads. This serves as a useful quality control metric for individual experiments, whereby datasets with high swapping rates can be flagged and discarded to avoid generating misleading biological results. The swapping fraction calculated using our approach leverages the expression levels of all genes, and makes no assumptions about the distribution of biological gene expression (i.e., before barcode swapping) across a plate. This should provide a more robust estimate of the swapped fraction for each experiment and improve the accuracy of any subsequent computational correction^11^.

Unique dual indexing represents another experimental solution to barcode swapping^2^. Under this scheme, two unique barcodes are used for each sample in a multiplexed sequencing experiment. A single barcode swap will move reads to barcode combinations that are not used by any other sample, thus avoiding any mixing of libraries between samples. However, the need for unique indices greatly restricts the number of libraries that can be multiplexed for a given number of barcodes (see Supplementary Note 7 for scalability calculations). This is particularly problematic for single-cell studies where large numbers of cell libraries need to be multiplexed for efficient sequencing. To use unique dual indexing in such cases, a researcher must have a large number of available barcodes, which may not be practical.

### Barcode swapping in droplet scRNA-seq experiments

New single-cell RNA-seq protocols use microfluidic systems to massively multiplex library preparation by capturing individual cells in droplets^3,12^. These methods enable the efficient generation of thousands of single-cell libraries in a single experiment. Cell labelling is achieved by the incorporation of a cell barcode in the reverse transcription step that occurs in each droplet. Each cell barcode is selected randomly from a large pool of possible sequences. A single sample barcode is then used to label different batches of single cells for multiplexed sequencing. The cell barcode is never free in solution; only the sample barcode is expected to swap.

We consider two major effects of barcode swapping in droplet-based experiments. Firstly, it is possible that the same cell barcode is used in two or more multiplexed samples. Between these samples, swapping will mix transcriptomes of different cells labelled with the same cell barcode, similar to the effect observed in plate-based assays. The second effect arises when a “donor” sample contains a cell barcode that is not present in another “recipient” sample. Swapping of molecules labelled with this donor-only barcode will produce a new artefactual cell library in the recipient sample. This new library will have a similar expression profile to the original cell in the donor sample and may be identified as a real cell. Indeed, swapping from cell libraries that are especially large may generate artefactual libraries in recipient samples that are as large and complex as real cells, making it difficult to find and remove them.

We demonstrated the existence of artefactual cell libraries in real data by testing whether samples from droplet-based experiments shared more cell barcodes than expected by chance. We obtained 10x Genomics data for human breast tumour cells and mouse epithelial cells, sequenced separately on the HiSeq 4000. In both of these experiments, at least one sample comparison exhibited excess sharing according to a hypergeometric test (Methods and Supplementary Figs. 31,32, Supplementary Note 5). We also obtained 10x Genomics data for mouse embryonic cells sequenced on the HiSeq 2500, and resequenced the aforementioned mouse epithelial cells on the HiSeq 2500. In both of these experiments, no excess sharing was observed (Supplementary Figs.  29,30). This is again consistent with an increased rate of barcode swapping on the new Illumina machines.

### Removing effects of barcode swapping in droplet experiments

**Fig. 3:**
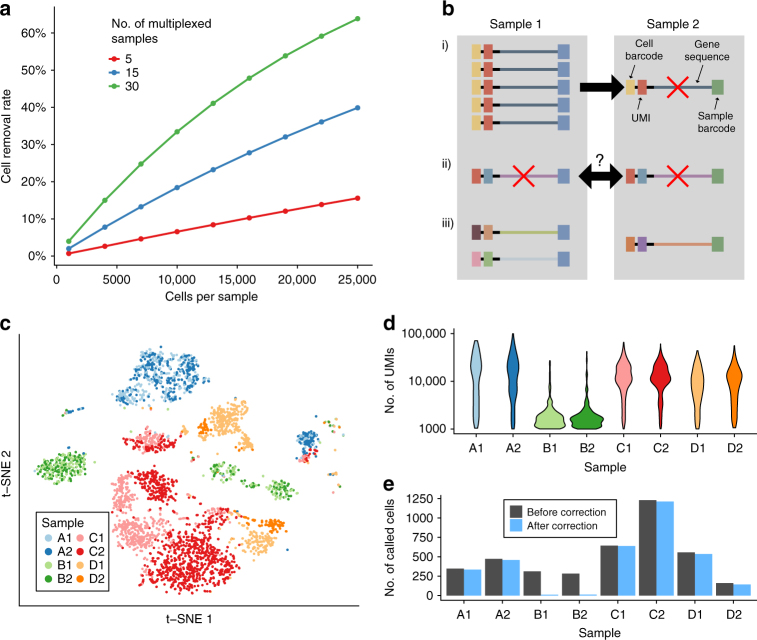
Characterization of barcode swapping in droplet-based scRNA-seq experiments. **a** The expected number of cells with shared cell barcodes in 10x Genomics samples that have been multiplexed for sequencing, for different numbers of samples and different numbers of captured cells per sample. The cell exclusion approach for barcode swapping would remove these cells. **b** A schematic of our method to remove swapped reads from droplet data. Reads found in different samples with the same combination of UMI, cell barcode, and aligned gene were considered to have swapped. If most reads (≥80%) were present in one sample, we excluded the molecule from all other samples (i). If reads were more evenly spread across samples, we excluded the molecule from all samples (ii). Reads in one sample only were retained (iii). **c** *t*-SNE plot of the expression profiles of mouse epithelial cells^17^. Each point represents a cell that is coloured by sample. Letters correspond to different experimental conditions while numbers represent biological replicates. **d** The distribution of the library sizes for called cells in each sample. Cells were called using emptyDrops, with an FDR threshold of 1% and a minimum of 1000 UMIs. **e** The number of called cells for each sample, before and after application of our swapped read exclusion algorithm

One obvious solution to mitigate the effect of barcode swapping is to discard any cell libraries with shared cell barcodes across multiplexed samples. This removes both homogenised and swap-derived artefactual libraries from further analysis, thus avoiding misleading conclusions driven by barcode swapping. However, cell-based removal is not appropriate when many cells are captured across many samples for a single multiplexed sequencing run. This is because many cells will share cell barcodes by chance, even in the absence of barcode swapping. Removal of these cells will result in unnecessary loss of data (Fig. 3a). For example, applying this strategy to 30 multiplexed samples of 20,000 cells each would exclude over 50% of cell libraries. An alternative approach is necessary for high-throughput droplet scRNA-seq datasets that are now routinely generated^4,5,13^.

We have developed a computational method that removes individual swapped reads from 10x Genomics data, avoiding the exclusion of entire cell libraries. Specifically, we considered molecules across multiplexed samples that contain the same combination of unique molecular identifier, cell barcode, and aligned gene. Due to combinatorial complexity, these molecules are extremely unlikely to arise by chance, and are almost certain to be generated by barcode swapping. For each observed combination of these labels, we calculated the fraction of reads that were present in each sample. Where one sample contained the majority of all reads for a molecule (≥80%), we considered this as the sample-of-origin, and removed the molecule count from all other samples. Where this was not the case, we removed the molecule from all samples (Fig. 3b), as an unambiguous determination of the sample-of-origin was not possible.

We applied our method to the aforementioned mouse epithelial cell dataset sequenced on the HiSeq 4000. In one experiment, two samples (B1, B2) appeared to contain many cells with expression profiles that were distinct from those of the cells in the other samples (Fig. 3c). We observed that cells in these samples had smaller library sizes than in other samples (Fig. 3d). Further inspection revealed that many cell barcodes in B1 and B2 were also present in other samples (Supplementary Note 5). We hypothesised that the majority of cell libraries in samples B1 and B2 derived from barcode swapping. Exclusion of swapped reads resulted in the loss of nearly all called cells from these samples (Fig. 3e, Methods and Supplementary Note 6), indicating that they consisted almost entirely of artefactual swapped libraries.

These results demonstrate the importance of excluding swapped reads prior to further analysis. Failure to do so would have resulted in misleading biological conclusions if the artefactual cells were used in analyses such as clustering and detection of differentially expressed genes. Indeed, the artefactual cells form their own cluster (Fig. 3c, Supplementary Fig. 33), and could be misinterpreted as a cell type exclusive to samples B1 and B2. We also observed that 2.5% of cell libraries from the other samples were no longer called as cells after removal of swapped reads. While only a small number of cells are removed from these samples, this may still be important in studies involving rare cell types where the presence of a few cells can affect the interpretation of the results.

As a control, we applied our method to two 10x Genomics experiments using mouse epithelial cells that were not multiplexed together. These were the aforementioned HiSeq 2500-sequenced mouse epithelial cells and the data from a similarly HiSeq 2500-sequenced published study^14^. Here, our method removed a negligible number of molecules (<0.0005%, Supplementary Note 6). This demonstrates that our method is able to specifically exclude swapped reads. Our method is implemented in the *DropletUtils* Bioconductor package for 10x Genomics experiments, and can be easily applied in a conventional analysis pipeline.

## Discussion

Using plate-based scRNA-seq datasets, we have reproducibly estimated the fraction of barcode-swapped reads on the HiSeq 4000 to be approximately 2.5%, which is lower than previously reported^1^. Different amounts of free DNA barcode did not affect our swapping fraction estimates, suggesting that free barcode concentration is not the primary factor determining the variation in barcode swapping rates across experiments. We recommend that plate-based scRNA-seq experiments that reuse cell barcodes should continue to be sequenced on non-patterned flow-cell machines such as the HiSeq 2500 to minimise barcode swapping. We have also implemented a computational method for removing swapped reads from 10x Genomics data without removing entire cell libraries. This permits the cost-effective use of the highest-throughput sequencing machines (e.g., the HiSeq 4000) for large-scale droplet scRNA-seq experiments while avoiding the confounding effects of barcode swapping.

## Methods

### Richard dataset analysis

Two 96-well plates of single-cell RNA-seq libraries of mouse T-cells were prepared according to the Smart-seq2 protocol^6^ with minor modifications (see Supplementary Note 3 for more information). Libraries were sequenced on both the HiSeq 2500 and the HiSeq 4000. Demultiplexing was performed allowing for reads to be assigned to libraries with both expected and “impossible” barcode combinations. Reads were mapped to the mm10 genome, and the number of read pairs mapping to the exonic region of each gene were counted.

We performed a simple linear regression on the number of observed swapped reads for each impossible combination, with respect to the number of reads available for swapping. Available reads are those in expected barcode combinations that share exactly one barcode with the impossible barcode combination. We used the gradient of the fitted line to obtain an estimate of the fraction of swapped reads for this experiment (see Supplementary Note 3). As a control, we repeated this procedure with the same libraries sequenced on HiSeq 2500.

### Nestorowa dataset analysis

We obtained count matrices from the authors of the Nestorowa et al. study^10^ (also available at NCBI GEO accession GSE81682). For each plate, we fitted a linear model to quantify contributions of different cells in the HiSeq 2500 data to the swapping-affected transcriptomes of the HiSeq 4000 data (see Supplementary Note 4). The fitted model was used to estimate the swapping fraction for that plate. The mean and standard deviation of swapping fractions were then computed across plates.

To quantify the concentration of free barcode, we used the BioAnalyzer Expert software on the traces collected during library preparation, considering the area under the curve between DNA lengths 45 to 70 base pairs. To quantify the concentration of sequencable cDNA, we considered the area under the curve between DNA lengths 400 to 800 base pairs. We then tested for any significant relationship between these concentrations and the estimated swapping fraction on each plate.

We fitted a gene-wise linear model to describe the contribution of the HiSeq 2500 expression profiles to those of the HiSeq 4000 data. This model differs from the previous one as it yields gene-specific estimates rather than entire plate estimates (see Supplementary Note 4). We tested for a non-zero swapping term in this model, and counted the number of genes with a significant positive or negative swapping term. As a control, we repeated this procedure on libraries sequenced on two lanes of a HiSeq 2500^15^.

### Droplet methods

Mouse epithelial cell libraries were generated as described in Bach et al.^14^.

We tested for an excessive sharing of cell barcodes using a hypergeometric test (see Supplementary Note 5). This was performed on data from both the HiSeq 4000 and, as a control, the HiSeq 2500.

To evaluate the cell exclusion strategy, we simulated the random sampling of 10x Genomics cell barcodes into each of a number of samples (see Supplementary Note  6). We applied cell exclusion by removing all barcodes that were shared between samples, and counted the fraction of cell libraries that were incorrectly discarded. This was repeated using different numbers of samples and different numbers of cells per sample.

We performed a simple analysis of the HiSeq 4000-sequenced mouse epithelial cells using the *R* packages scran^16^ and Rtsne^17^ (see Supplementary Note 5).

Our molecule exclusion approach was implemented by removing molecules that share the same combination of UMI, cell barcode, and aligned gene (see Supplementary Note 6). This method is available in the DropletUtils package. We tested the method on the HiSeq 4000-sequenced mouse epithelial cell dataset, calling cells using emptyDrops^18^ using an FDR threshold of 0.01 and a minimum cell size of 1000 UMIs.

### Code availability

A Github repository (https://github.com/MarioniLab/BarcodeSwapping2017) contains a detailed report that expands on the analyses described herein, describing models and showing results. The repository also contains a script to download the processed data, and the code used to generate the report.

### Data availability

Raw data can be acquired from ArrayExpress accession codes E-MTAB-6843 for plate-prepared mouse T-cells, and E-MTAB-6854 for droplet-prepared HiSeq 2500- and HiSeq 4000-sequenced mouse epithelial cells.

## Electronic supplementary material

Supplementary Information
