# Supplementary material for: Detection and removal of barcode swapping in single-cell RNA-seq data
Source: Nat Commun. 2018 Jul 10;9:2667. doi: 10.1038/s41467-018-05083-x (PMC6039488; doi:10.1038/s41467-018-05083-x)
Supplement: Supplementary file 1 — Supplementary Information [file 41467_2018_5083_MOESM1_ESM.pdf]

Detection and removal of barcode swapping in single-cell RNA-seq data

Supplementary Information

Griffiths *et al.*

Supplementary material: Detection and removal of barcode swapping from single-cell RNA-seq data

***Jonathan A. Griffiths*[1], *Arianne C. Richard*[1,2], *Karsten Bach*[3], *Aaron T.L. Lun*[1] and *John C. Marioni*[1,4,5]**

[1]Cancer Research UK Cambridge Institute, University of Cambridge, CB2 0RE, United Kingdom

[2]Cambridge Institute for Medical Research, University of Cambridge, Cambridge, CB2 0XY, United Kingdom.

[3]Department of Pharmacology, University of Cambridge, CB2 1PD, United Kingdom

[4]EMBL European Bioinformatics Institute (EMBL-EBI), Wellcome Genome Campus, CB10 1SD, Hinxton, United Kingdom

[5]Wellcome Trust Sanger Institute, Wellcome Genome Campus, CB10 1SA, Hinxton, United Kingdom

15/05/2018

The latest version of this document can be found on the Marioni Lab GitHub page (https://github.com/MarioniLab/BarcodeSwapping2017). That version also includes expandable code chunks. The repository also contains all code used for analysis, and instructions to obtain the raw data required to reproduce the results shown below.

Supplementary Note 1: Introduction

With the rapid increase in throughput of next-generation sequencing technologies, an individual run of a typical sequencing machine (such as those produced by Illumina) generates many more reads than is necessary for interrogating single libraries generated by most functional genomic assays. To make efficient use of these machines, DNA libraries are typically pooled together prior to sequencing, in a process known as “multiplexing”. Briefly, unique barcodes are ligated onto the ends of the DNA molecules within each library before pooling. This incorporates a known sequence into each read, allowing the assignment of reads to their libraries of origin after sequencing. Multiplexing also ensures that technical effects are consistent across samples, avoiding batch effects between sequencing lanes or flow cells; and can provide robustness against the failure of sequencing lanes, which would otherwise result in the loss of entire samples. As such, multiplexing is widely

considered to be standard practice for many sequencing experiments, and is essential for cost-effective analysis of small libraries such as those in single-cell RNA sequencing (scRNA-seq) studies.

The most recent DNA sequencing machines released by Illumina (HiSeq 3000/4000/X, X-Ten, and NovaSeq) use patterned flow cells to improve throughput and cost efficiency. On these new flow cells, the process of "seeding" DNA molecules into the patterned wells and amplification of the seeded DNA occur simultaneously. These machines have been in use for several years in a diverse range of genomic fields. However, it has been recently reported that the use of these machines can lead to the mislabelling of DNA molecules with the incorrect library barcode (Sinha et al. 2017). The mislabelling is likely driven by the extension of free barcode molecules using other DNA molecules as a template (Supplementary Figure 1). The phenomenon has been acknowledged by Illumina (Illumina 2017), although estimates of swapping fractions vary between reports (Costello et al. 2017). It is unclear whether a permanent solution to the problem will be forthcoming as rapid amplification after seeding is critical to the operation of the patterned flow cell machines (Sinha et al. 2017).

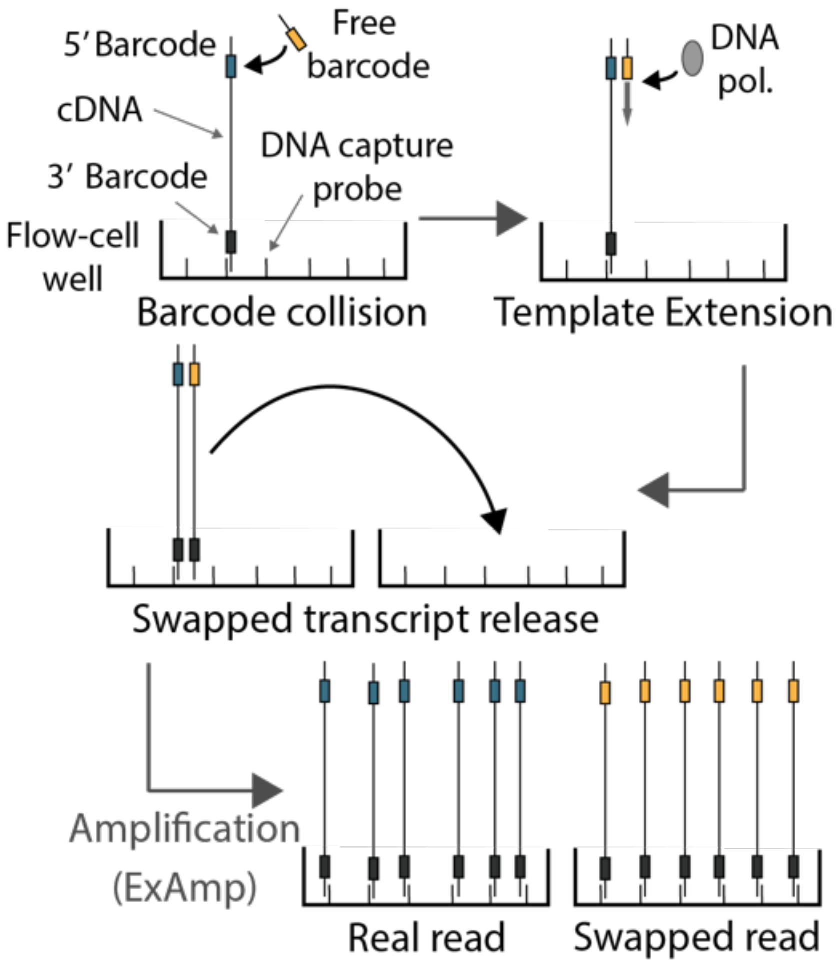

Supplementary Figure 1: Schematic of the mechanism of barcode swapping on the HiSeq 4000, as proposed by Sinha et al (2017).

This "swapping" of barcode labels (also called "hopping" or "switching") is problematic for analyses of sequencing data. Reads labelled with a barcode specific to a given sample may have originated from any other multiplexed sample in the same pool, compromising the interpretation of the sample labels and their use in downstream analyses. This phenomenon is particularly relevant for single-cell -omics assays, where a large number of samples (i.e.,

cells) are necessarily multiplexed together for efficient use of sequencing resources. Our manuscript (*"Detection and removal of barcode swapping in single-cell RNA-seq data"*) quantifies the effect of barcode swapping in a variety of single-cell RNA-seq datasets. We show that swapping can create artefactual cell libraries in droplet-based scRNA-seq experiments. Finally, we have implemented a strategy to eliminate the effects of barcode swapping in droplet-based datasets without excessive loss of data.

Supplementary Note 2: Definition of terms

Before continuing, we define a few terms:

- Swapping occurs between a **donor** library, from which the transcript originated, and **recipient** libraries, in which the swapped read is detected after sequencing.
- The **swapping fraction** is defined as the fraction of reads that have been mislabelled, from the set of all cDNA-derived reads in a pool of multiplexed libraries sequenced on a single flow cell lane.

Supplementary Note 3: Plate-based analysis of the Richard data

Description of the experimental design

We consider two 96-well plates of single-cell RNA-seq libraries for mouse T-cells. We used dual indices for cell labelling, i.e., a different barcode was used at each end of the molecule. The barcodes used for each plate are from mutually exclusive sets - any barcode from one plate was never used on the other (Supplementary Figure 2). For sequencing, all cell libraries from the two plates were multiplexed.

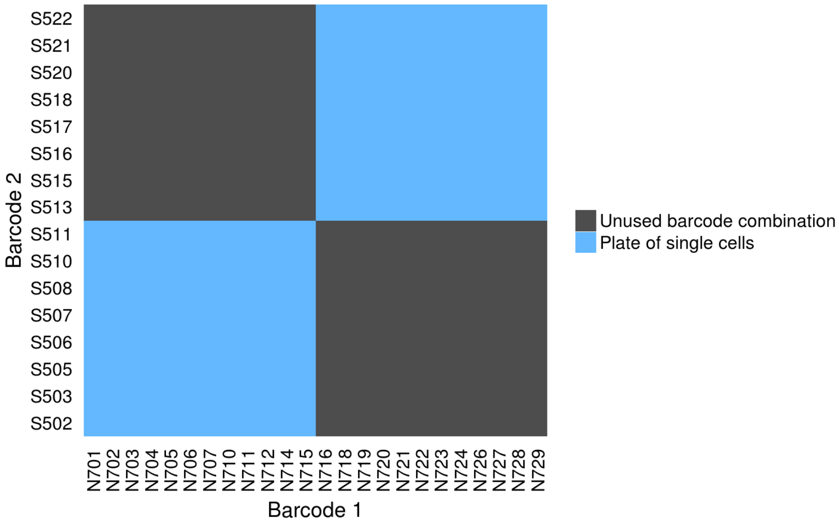

Supplementary Figure 2: Overview of the experimental design in the Richard dataset. Each position of the plot represents a barcode combination. Each of the blue blocks represents at 96-well plate. One barcode combination (N729,S522) in one of the plates did not contain a cell, but did contain barcodes and spike-in transcripts. Barcode combinations in the grey positions were not used, and thus should not contain sequencing reads.

Details of data generation

Cells were prepared for single-cell RNA-seq using the SmartSeq2 protocol (Picelli et al. 2014), adapted as described in (Richard et al. 2018). Briefly, cells were sorted into 96-well plates with 4 μL of lysis buffer: 0.11 % (v/v) Triton X-100 (Sigma), 12.5 mM DTT (Thermo Fisher Scientific), 2.5 mM dNTP mix (Thermo Fisher Scientific), and 2.3 U SUPERase In RNase inhibitor (Thermo Fisher Scientific). Annealing mix, composed of diluted ERCC RNA Spike-In Mix (Thermo Fisher Scientific) and 10 μM oligo-dT30VN (Sigma), was added 1 μL per well, and reverse transcription was performed using SuperScript II (Invitrogen). cDNA was amplified (23 PCR cycles) and purified with Ampure XP Beads (Agencourt) at 0.7 beads / 1 DNA (v/v). Library preparation was performed with the Nextera XT DNA Sample Preparation Kit using indexes from the Nextera XT Index Kit v2 Set A and Set D (Illumina). Libraries from each plate were pooled and purified with Ampure XP beads before quantification with the KAPA Library Quantification Kit (Roche). Library pools from each plate were combined in equimolar quantities. Library sequencing was performed both on an Illumina HiSeq4000 and an Illumina HiSeq2500.

Reads were demultiplexed allowing for any of the barcode combinations shown in Supplementary Figure 2 (including the impossible combinations). Read mapping was performed using the *Subread* aligner (v1.5.1) (Liao, Smyth, and Shi 2013) to the mm10 build of the mouse genome with additional ERCC sequences (http://www.thermofisher.com/order/catalog/product/4456739 (http://www.thermofisher.com/order/catalog/product/4456739)). We used a Phred offset of 33 and only considered uniquely mapped reads, with default values for all other parameters. We counted the number of reads mapped to each gene in each cell using the `featureCounts` function (Liao, Smyth, and Shi 2014) in the *Rsubread (http://bioconductor.org/packages/Rsubread)* package with default options (except for `minMQS=10`, to retain only high-quality alignments). This function assigned reads to exonic regions of each gene in the Ensembl mm10 annotation (version 82) or to ERCC spike-in transcripts.

Examination of the library sizes

In the absence of barcode swapping, it should be impossible to observe mapped reads with barcodes from each of the two different plates, i.e., there should be no mapped reads in the grey areas of Supplementary Figure 2. We will refer to these barcode combinations as “impossible” combinations, in comparison to the expected combinations in orange. For the expected combinations where cells have been loaded, there are many mapped reads (Supplementary Figure 3) as expected. In the impossible barcode combinations, we observe a lower but non-zero number of mapped reads, consistent with the presence of barcode swapping.

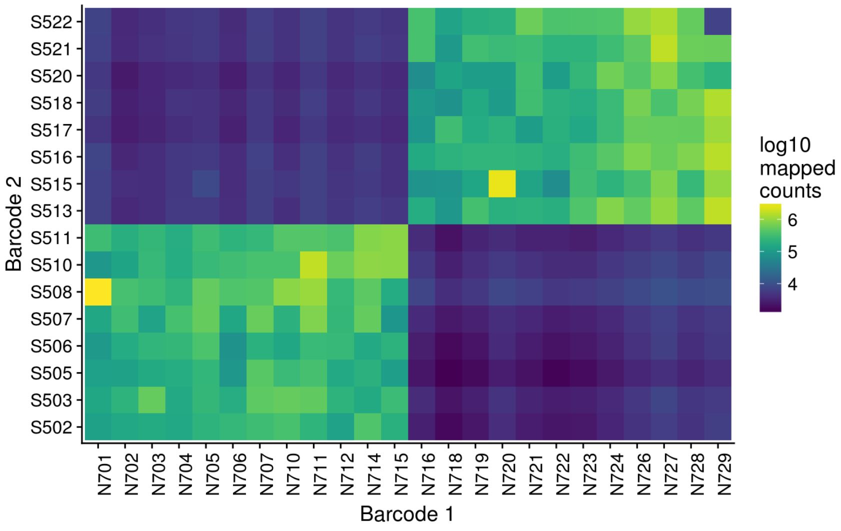

Supplementary Figure 3: Number of mapped reads per barcode combination, coloured on a $\log_{10}$ scale.

The distribution of total number of mapped reads (i.e., library sizes) for all combinations are shown in Supplementary Figure 4 and Supplementary Figure 5. The impossible combinations have a median mapped-read library size that is 1.5% of the median size of the expected combinations. The total number of mapped reads assigned to impossible combinations is 1.1% of that assigned to expected combinations. Note that the empty well is still considered as an expected barcode combination due to the presence of ERCC spike-in transcripts.

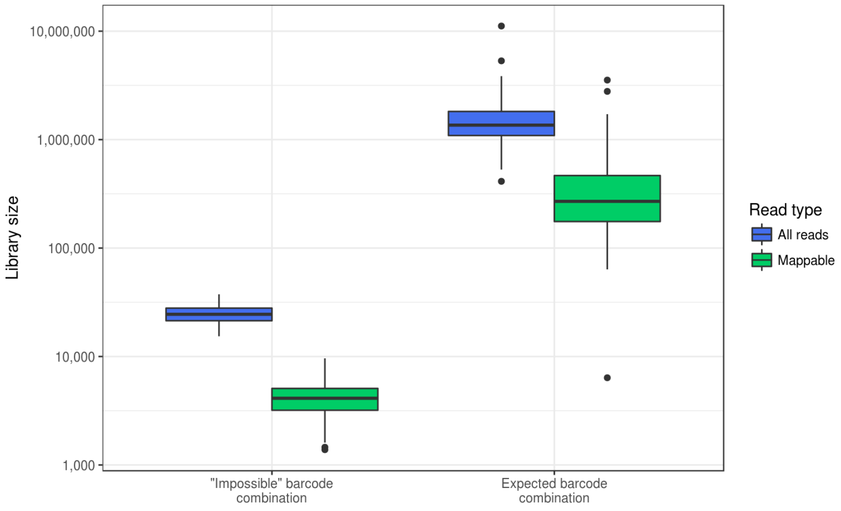

Supplementary Figure 4: Boxplots of the total number of reads for the impossible and expected barcode combinations. Dots represent barcode combinations that have totals more than 1.5 interquartile ranges from the edge of the box.

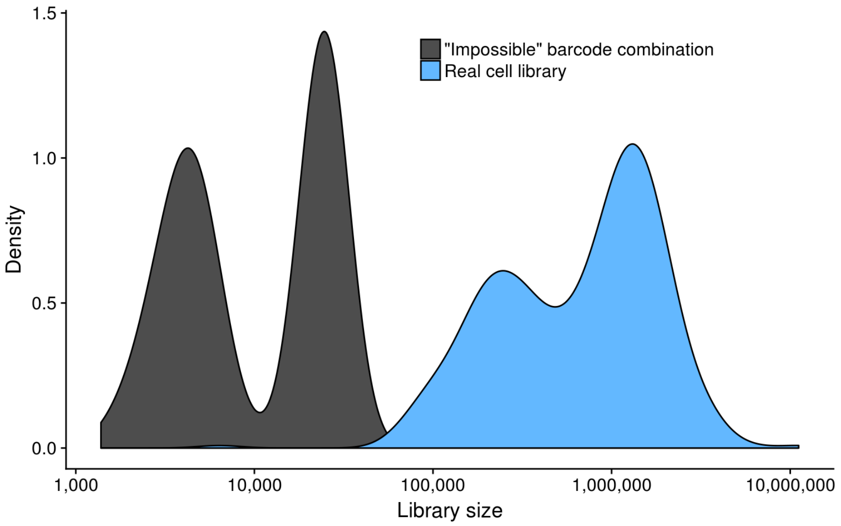

Supplementary Figure 5: Distribution of library sizes for all expected and impossible barcode combinations.

We focused on mapped reads as these are most relevant to downstream analyses. Nonetheless, we observe similar results for all reads, consistent with the ability of barcode swapping to affect all molecules on the flow cell. The median number of all reads assigned to an impossible combination is 1.8% of that assigned to an expected combination, while the total number of reads assigned to impossible combinations is 1.6% of the total number of reads assigned to expected combinations.

We emphasize that our results are highly robust to contamination from ambient RNA or human/bacterial sources. Regardless of the amount of contamination, the impossible barcode combinations should not contain any reads, because these pairs of barcodes were never mixed in library preparation. Barcode swapping is the only possible mechanism for obtaining a substantial number of non-zero reads for these combinations. (We ignore the possibility of barcode sequencing errors causing misassignment of reads, which would be extremely unlikely for 8 bp barcodes that are well separated in base space.) This provides a point of difference for our experimental design compared to that of Sinha et al. (2017), who estimated the swapping rate based on empty wells that still contained barcodes. In their design, contamination would result in mapped reads in the empty wells and the appearance of an elevated rate of swapping.

Swapping fraction estimation on the HiSeq 4000

Denote each barcode combination as (i, j) where barcode $i \in 1, \dots, 16$ represents a row in Supplementary Figure 2 (with $i = 1 \Rightarrow \text{S522}, i = 16 \Rightarrow \text{S502}$) and barcode $j \in 1, \dots, 24$ represents a column ($j = 1 \Rightarrow \text{N701}, j = 24 \Rightarrow \text{N729}$). Let $M_{i,j}$ denote the number of seeded cDNA molecules that truly originate from this combination, and let $X_{i,j}$ denote the number of mapped reads. $M_{i,j}$ therefore represents the true source of reads, while $X_{i,j}$ represents the reported source after swapping. Impossible barcode combinations are those with $1 \leq i \leq 8, 1 \leq j \leq 12$ or $9 \leq i \leq 16, 13 \leq j \leq 24$, and have $M_{i,j} = 0$ by definition.

We assume that barcode swapping is rare, so it is unlikely that one molecule will undergo more than one round of swapping. This means that reads will only be transferred between combinations that already share a single barcode. This

is illustrated in Supplementary Figure 6. For that example, the combination `N719` and `S505` (red position) would receive swapping contributions from the cells in the blue positions.

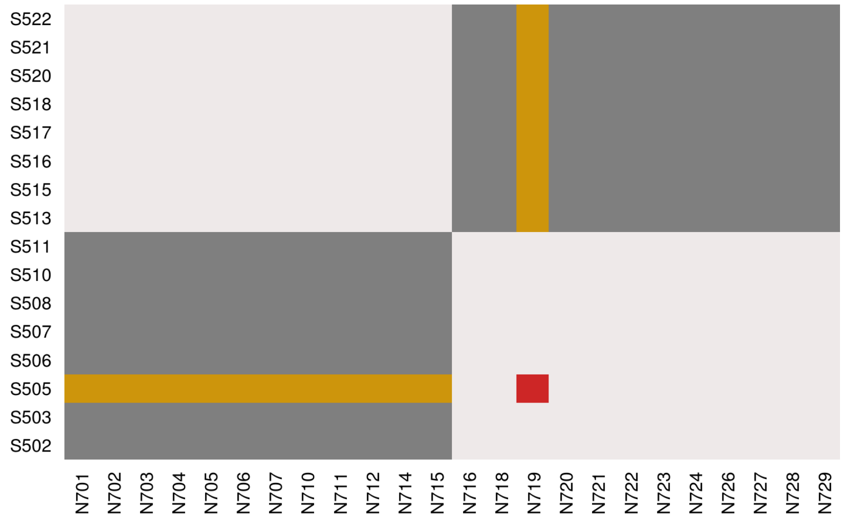

Supplementary Figure 6: A schematic of the expected barcode combinations that are potential donor libraries (orange) for swapping into a recipient library (red), an impossible combination (S505/N719). Only a single barcode needs to be swapped for transcripts in the blue combinations to appear as reads in the red combintion. Used barcode pairs are shown in grey, and unused barcode pairs are shown in black.

Let τ be the conversion rate of seeded cDNA molecules to mapped reads in the same cell library. This is probably less than 1 due to the presence of unmappable sequences (e.g., transcribed repeats). Moreover, a seeded PCR duplicate of a cDNA molecule (formed on the flow cell) would normally count as a new read for the gene in the original cell. However, if the duplicate molecule has swapped its barcode, the cell has effectively "lost" this additional read. This will further decrease the value of τ.

We further assume that the number of observed swapped reads is proportional to the number of molecules that are available for swapping. Define ρ as the rate of swapping from any single donor library to any single recipient library, i.e., the proportion of molecules in the donor library that appear as mislabelled reads in the recipient. For each barcode combination, the number of reads can be modelled as

$$\tilde{X}_{i,j} = \tau M_{i,j} + \rho \left(\sum_{k \neq j} M_{i,k} + \sum_{l \neq i} M_{l,j} \right) .$$

As barcode swapping is rare, ρ should be very low such that $\tilde{X}_{i,j} \approx \tau M_{i,j}$ for the expected barcode combinations where $M_{i,j} > 0$. We further approximate $\tilde{X}_{i,j}$ for these expected combinations by replacing it with the observed $X_{i,j}$. This means that, for each impossible combination (i^*, j^*), we have

$$\tilde{X}_{i^*,j^*} \approx \frac{\rho}{\tau} \left(\sum_{k \neq j^*} X_{i^*,k} + \sum_{l \neq i^*} X_{l,j^*} \right) .$$

This represents a linear relationship between the library size for each impossible combination and the sum of the library sizes for all expected combinations with which it shares a single barcode. We estimate the parameters of this relationship by fitting a line to each X_{i^*,j^*} against the corresponding sum using ordinary least squares, as shown in Supplementary Figure 7.

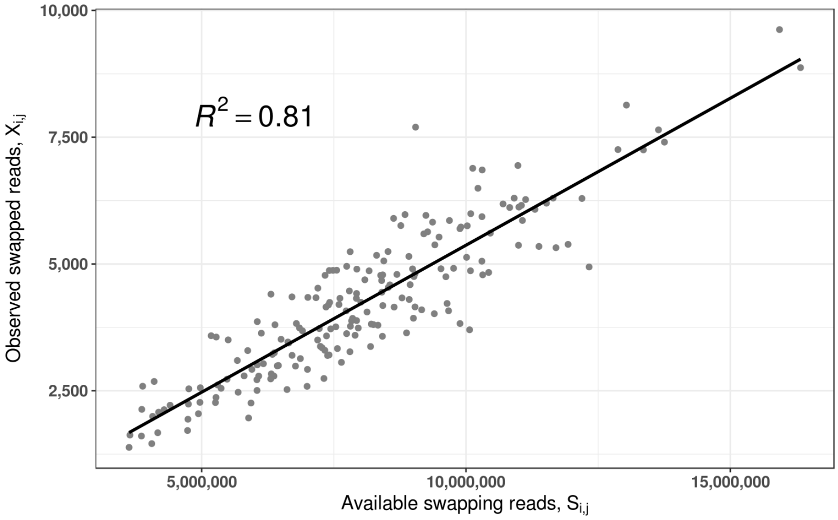

Supplementary Figure 7: Relationship between the library size for each impossible combination and the sum of library sizes for all expected combinations sharing a single barcode in the HiSeq 4000 data. Each point represents an impossible barcode combination. The line of best fit is shown along with the coefficient of determination.

We estimate the total number of mislabelled reads across all combinations to be

$$\rho \sum_{i=1}^{16} \sum_{j=1}^{24} \left(\sum_{k \neq j} M_{i,k} + \sum_{l \neq i} M_{l,j} \right)$$

$$= 38\rho \sum_{i=1}^{16} \sum_{j=1}^{24} M_{i,j}$$

$$= 38\rho \sum_{(i,j) \in \mathcal{E}} M_{i,j}$$

$$\approx \frac{38\rho}{\tau} \sum_{(i,j) \in \mathcal{E}} X_{i,j}$$

where $\mathcal{E}$ is a set of all expected combinations. The multiplication by 38 is due to the fact that there are 38 available destinations for a single-barcode-swapped read from any single expected combination (Supplementary Figure 8). In other words, we sum each $M_{i,j}$ 38 times in the first line of the above expression. This includes the impossible barcode combinations as well as the real cell combinations.

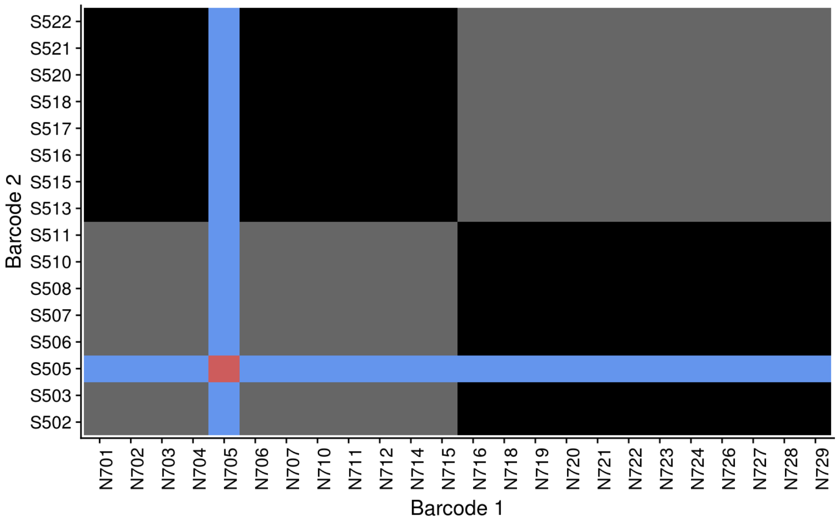

Supplementary Figure 8: Schematic illustrating the contribution of a donor library (red) to each of 38 recipient libraries that share a single barcode (blue). Cell-loaded combinations are shown in grey, and unloaded combinations are shown in black.

To obtain the swapping fraction in this experiment, we divide by the total number of mapped reads:

$$\frac{38\rho}{\tau}\left(\frac{\sum_{(i,j)\in\mathcal{E}} X_{i,j}}{\sum_{i=1}^{16}\sum_{j=1}^{24} X_{i,j}}\right)$$

This yields an estimated swapping fraction of 2.180 $\pm$ 0.0765%. Notably, this is higher than the median-to-median fraction of 1.5%. This is because the median-to-median fraction only considered swapped reads in the impossible barcode combinations, whereas this slope-estimated value considers reads that swap across the entire set of barcode combinations.

Note that we fitted the line in Supplementary Figure 7 with an intercept term. The value of the intercept is -427.5 $\pm$ 170.0. This is close to zero compared to a median library size of 4123 for the impossible combinations, consistent with our model for $\tilde{X}_{i^*,j^*}$.

Swapping fraction estimation on the HiSeq 2500

The exact same pool of multiplexed libraries was also sequenced on a HiSeq 2500. This provides a negative control dataset where barcode swapping should not be present (or, at least, present at a lower rate than encountered in the HiSeq 4000). We see strong correlation between library sizes from the two machines, as shown in Supplementary Figure 9.

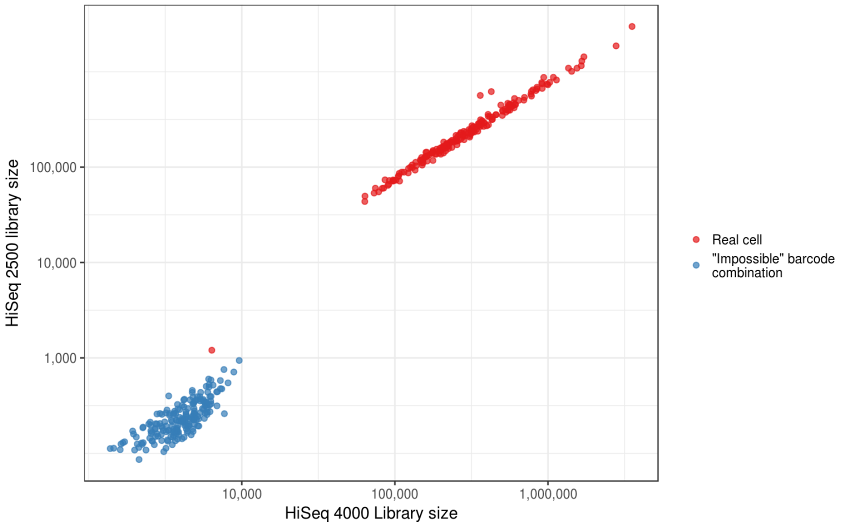

Supplementary Figure 9: Library sizes for all expected (red) and impossible (blue) barcode combinations in the HiSeq 2500 and 4000 data. The expected combination with the small library size corresponds to the empty well.

In the HiSeq 2500 data, many fewer reads are present in the impossible barcode combinations. The impossible combinations have a median library size of 0.11% the median size of the expected combinations (compared to 1.5% from the HiSeq 4000). Considering all mapped reads on the plate, there are 0.082% as many in the impossible combinations as in the expected ones (compared to 1.1% from the HiSeq 4000).

We applied the same model to the HiSeq 2500 data as described above for the HiSeq 4000 data. Results are shown in Supplementary Figure 10.

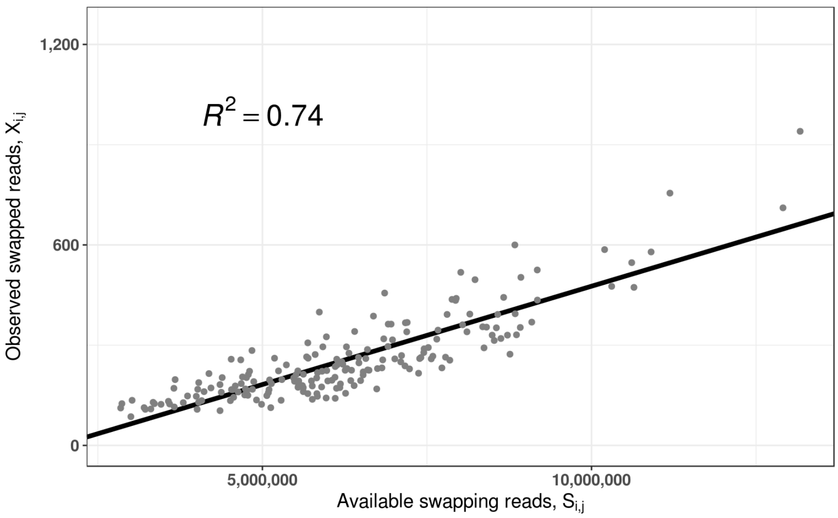

Supplementary Figure 10: Relationship between the library size for each impossible combination and the sum of library sizes for all expected combinations sharing a single barcode in the HiSeq 2500 data. The line of best fit is shown along with the coefficient of determination.

Interestingly, we still observe the swapping pattern in the HiSeq 2500 data. However, the estimated swapping fraction is approximately an order of magnitude lower: 0.223 $\pm$ 0.00955% on the HiSeq 2500, compared to 2.180 $\pm$ 0.0765% on the HiSeq 4000.

Supplementary Note 4: Plate-based analysis of the Nestorowa data

Dataset overview

To confirm the existance of barcode swapping, we used data from another published study (Nestorowa et al. 2016), which we refer to as the Nestorowa data. 16 sets of single-cell RNA-seq libraries were each generated on a 96-well plate, pooled together and sequenced on a single lane on a HiSeq 2500 machine. At a later date, each of the same pooled libraries were sequenced on a HiSeq 4000. For each plate, exactly the same pool of libraries was used in both sequencing runs, so the only differences between the results should be caused by the sequencing machines and Poisson sampling noise (J. C. Marioni et al. 2008). This is important, because repooling of libraries may reduce the precision of our swapping fraction estimate, or introduce systematic confounding.

Examination of crosshair swapping patterns

We identified the gene with the largest read count in a single cell in the first plate of cells - *Igkc*. This gene is almost uniquely expressed in one cell in the plate. On both the HiSeq 2500 and 4000 machines, we observe a “crosshair” pattern of expression for this gene (i.e., along the row and column of the most highly-expressing cell), as shown in Supplementary Figures 11 and 12. This is the same pattern that was reported by Sinha et al. (2017) and is attributable to barcode swapping from a single donor cell library to all recipient libraries sharing a single barcode.

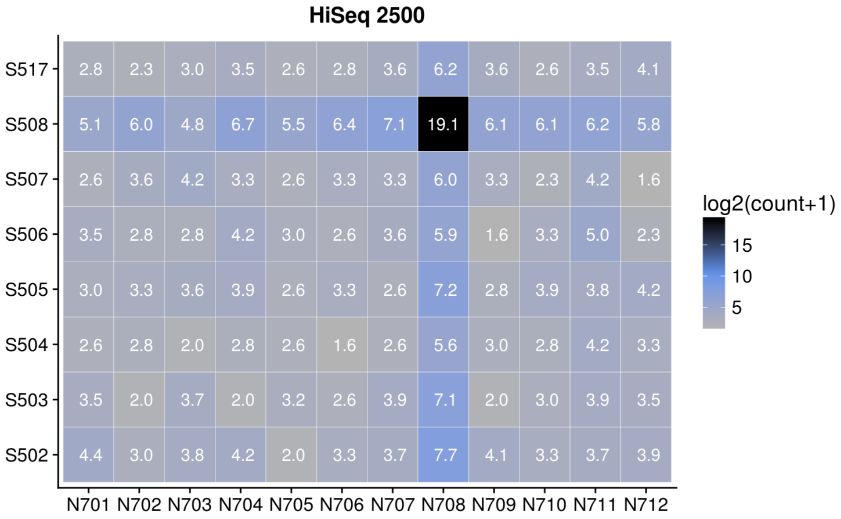

Supplementary Figure 11: Expression of the gene with the highest read count in any cell, across all cells on the first plate after sequencing on the HiSeq 2500.

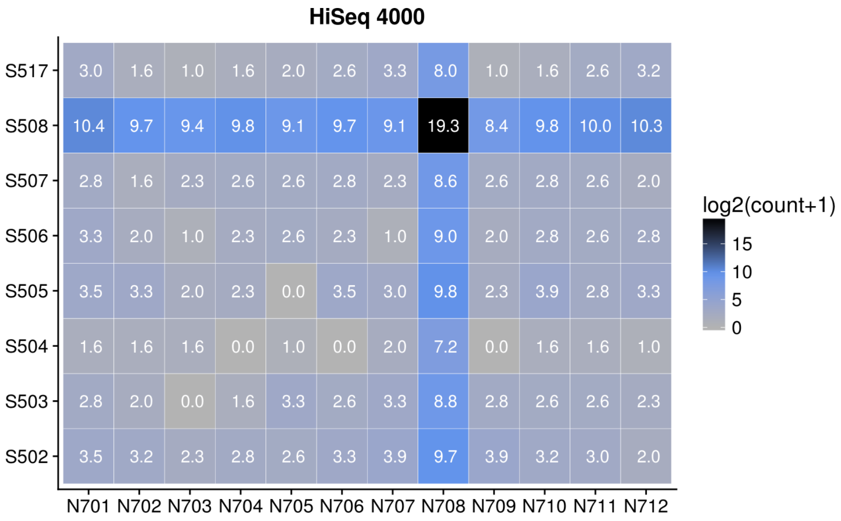

Supplementary Figure 12: Expression of the gene with the highest read count in any cell, across all cells on the first plate after sequencing on the HiSeq 4000.

While the crosshair pattern is present with both machines, it is clearly stronger on the HiSeq 4000. There are 1.93% as many *Igkc* reads in the crosshair as in the central highly-expressing cell in the HiSeq 4000 data, compared to 0.257% for the HiSeq 2500. This is consistent with the order-of-magnitude difference in the swapping fraction between the two technologies estimated from the Richard data. The increase in *Igkc* coverage in the crosshair with the HiSeq 4000 is clearly shown by visualizing the $\log_2$-fold change in coverage for each cell (Supplementary Figure 13).

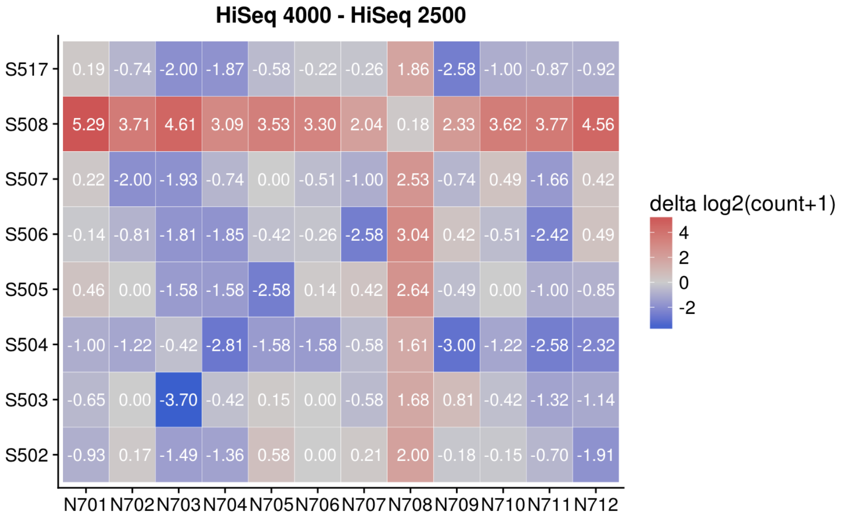

Supplementary Figure 13: $\log_2$-fold change of the read count of the *Igkc* gene in the HiSeq 4000 data over the HiSeq 2500 in every cell of the first plate. A pseudo-count of 1 was added to avoid undefined log-fold changes.

These crosshair patterns demonstrate that swapping along rows and columns is the primary mode of read transfer between libraries. This supports our assumption that swapping is a rare event. In the vast majority of cases, swapping will occur no more than once to the same molecule, restricting the transfer of reads to libraries that already share a single barcode.

Quantifying the swapping fraction

To quantify the swapping fraction, we assume that the HiSeq 2500 data contains negligible amounts of barcode swapping compared to the HiSeq 4000. This is motivated by the order-of-magnitude difference between the two sequencing machines observed in the Richard data. Our assumption allows us to treat the HiSeq 2500 data as an unbiased representation of the true expression profile for each cell, unaffected by swapping.

We applied a model that identifies contributions of different cells in the HiSeq 2500 data to the swapping-affected transcriptomes of the HiSeq 4000 data. Let Y_{4000} denote the $G \times C$ read count matrix for the HiSeq 4000 libraries, where rows are genes (for G total genes) and columns are cells (for C total cells). Let Y_{2500} denote the equivalent count matrix for the HiSeq 2500 libraries. Let R denote a $C \times C$ matrix representing the contribution of the HiSeq 2500 counts to the HiSeq 4000 counts. Each entry of R defines the proportion of one HiSeq 2500 library that makes up one HiSeq 4000 library. To illustrate, take the value at (c_1, c_2) in R, and multiply it by the number of reads for cell c_1 in the HiSeq 2500 data. This represents the number of reads from a cell c_1 in the HiSeq 2500 data that contributes to a cell c_2 in the HiSeq 4000 data.

For a cell with a given pair of barcodes, we need to discriminate between the contribution of other cells with exactly one shared barcode and other cells with no shared barcodes. To do this, let $R = R_0 + R_1 + R_2$ where each of R_0, R_1 and R_2 is a matrix of the same dimensions as R. The element (c_1, c_2) of each matrix is defined as

$$R_0[c_1, c_2] = \begin{cases} \gamma & \text{if cells } c_1 \text{ and } c_2 \text{ share no barcodes} \\ 0 & \text{otherwise} \end{cases}$$
$$R_1[c_1, c_2] = \begin{cases} \beta & \text{if cells } c_1 \text{ and } c_2 \text{ share exactly one barcode} \\ 0 & \text{otherwise} \end{cases}$$
$$R_2[c_1, c_2] = \begin{cases} \alpha & \text{if cells } c_1 \text{ and } c_2 \text{ share both barcodes} \\ 0 & \text{otherwise} \end{cases}$$

The value of α represents the contribution of each cell in the HiSeq 2500 data to the corresponding cell in the HiSeq 4000 data. This includes the “loss” of potential reads, i.e., PCR duplicates unaffected barcode swapping, as previously discussed for τ in the Richard analysis. The value of β captures the rate of row-column swapping from a donor cell c_1 to a recipient cell c_2, while γ captures swapping between barcode combinations that do not share any barcodes. In the terminology of the framework used for the Richard analysis, we are using the HiSeq 2500 read counts for each donor cell as a proxy for the number of molecules available for swapping in the HiSeq 4000 data. Finally, note that all terms can be globally scaled to capture differences in sequencing depth between the HiSeq 2500 and 4000 data.

We define the relationship between the two count matrices as:

$$Y_{4000} = Y_{2500} R + \epsilon$$

where ϵ represents the residual error. The aim is to obtain estimates of α, β, and γ for each plate, using information across many genes to stabilise the estimates.

Gene selection is important for the fitting of this model. We examine the expression of two genes on the first HiSeq 2500 plate in the dataset in Supplementary Figures 14 and 15.

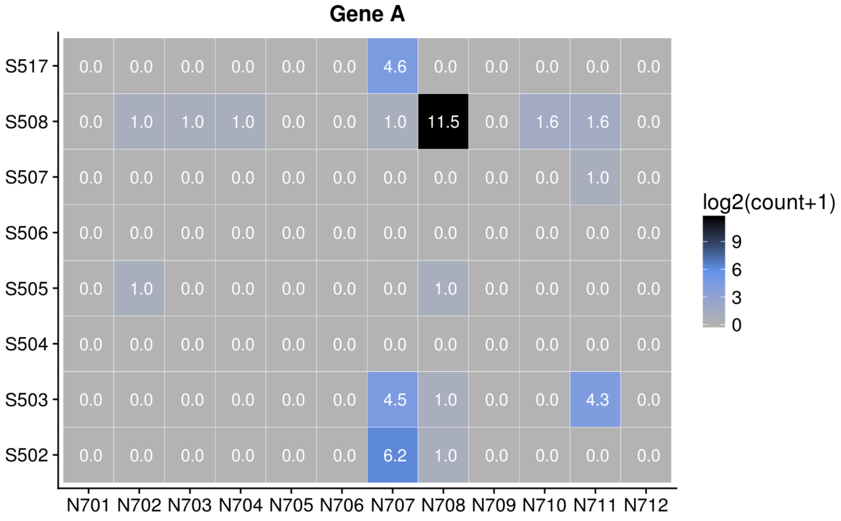

Supplementary Figure 14: Gene expression pattern of gene A, shown in log-counts for all cells on the first plate.

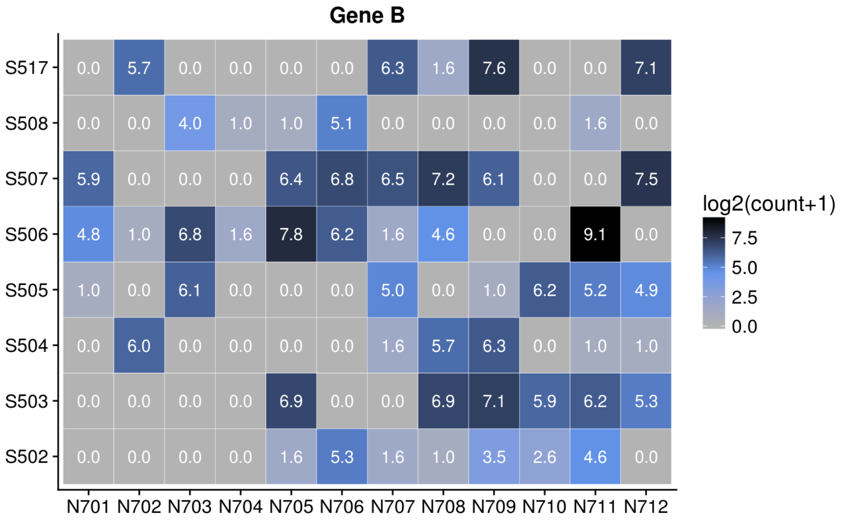

Supplementary Figure 15: Gene expression pattern of gene B, shown in log-counts for all cells on the first plate.

Both genes are expressed in only a subset of the cells on the plate, but gene B is expressed much more broadly than gene A. For gene B, it is harder for the model to distinguish between contributions from other cells on the row and column of a certain cell (β) or from all the other cells on the plate (γ), because many cells in both of these sets express the gene at high levels. By contrast, gene A is expressed at a high level in only very few cells, making it easier for the model to distinguish between large values of β and γ. Genes expressed broadly in the manner of gene B are therefore less informative for model fitting than those expressed like gene A.

To identify the most informative genes on a single plate, we define the "information score" for each gene as the ratio of the maximum expression value to its 90th percentile. This value will be highest when only a very few cells on the plate are highly expressing the gene. We only calculate this score for genes that are present at a minimum level of 500 counts in at least one cell in the HiSeq 2500 data. It is important to have a large number of transcripts present, otherwise swapping may be too rare to detect. The information scores are plotted in Supplementary Figure 16 for a few randomly chosen plates.

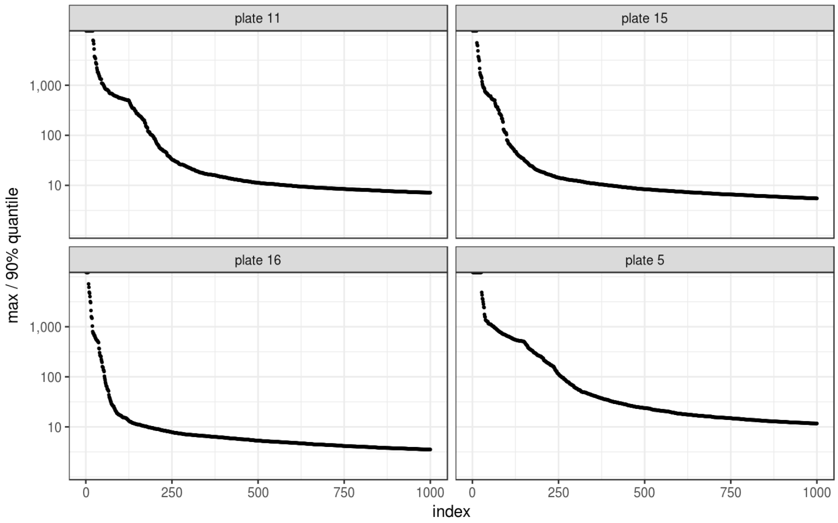

Supplementary Figure 16: Top 1000 genes by the highest information scores, ranked in descending order. Genes with infinite scores are shown as points at the top of the plots. Information scores are computed separately for each gene in each plate.

We identify the top 500 genes with the highest information scores and use only the corresponding rows in Y_{2500} and Y_{4000} for downstream model fitting. The informative gene set is selected separately for each plate. We consider 500 genes to ensure that we include the infinite scores (i.e., where the 90th percentile is 0) and to ensure that we do not exclude informative genes for other plates, which may have longer tails than the distributions shown above. We have also repeated the fits using the top 250 or 1000 genes, which yield similar estimates of the α, β and γ parameters (see below, Supplementary Figure 19). Note that the chosen subset of genes will differ between plates, but this does not affect the comparability of the parameter estimates between plates.

We emphasize that the maximum expression value on a plate does not substantially drive selection of genes (Supplementary Figure 17). This means that we are not simply selecting for genes with high maximum expression. Rather, we are identifying genes where the maximum expression value is a clear outlier relative to expression in other cells on the plate.

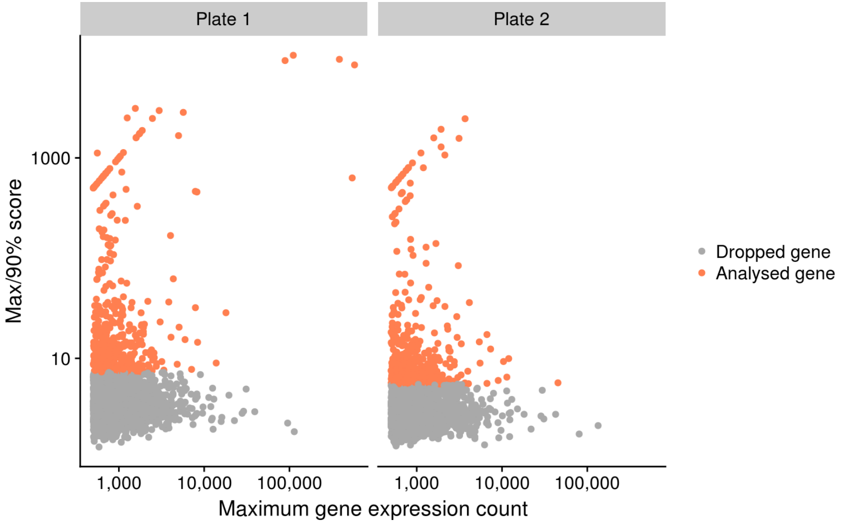

Supplementary Figure 17: Relationship between the maximum expression and the information score for each gene, using the first two plates in the dataset.

We used Poisson precision weights in our model to account for the mean-variance relationship of count data. Counts were weighted by the reciprocal of the square-root of their gene's mean expression, to ensure that the most highly-expressed genes do not dominate the least-squares fit. We fitted a constrained linear inverse model using the *limSolve (https://CRAN.R-project.org/package=limSolve)* package, to avoid obtaining negative values of α, β, and γ. Gene subsetting and fitting of the model was performed separately for each plate of cells, so each plate had its own informative gene set.

Across the 16 plates assayed, we acquired a distribution of estimates for each parameter in R. For example, we observe values between 0 and 0.00279 for the single shared barcode contribution term β.

To calculate the swapping fraction for each plate, we estimated the number of reads in the HiSeq 4000 libraries that were contributed from the HiSeq 2500 libraries via swapping. We include both single barcode swaps (R_1) and double barcode swaps (R_0) in this estimate. For simplicity, let us denote the summation of all elements $x_{i,j}$ of the matrix X as

$$\sigma(X) = \sum_i \sum_j x_{i,j}$$

The total number of swapped reads is

$$\sigma(Y_{2500}(R_0 + R_1))$$

We divided this by the total number of reads in the fitted model (i.e., $\sigma(Y_{2500}R)$) to obtain an estimate of the swapping fraction for each plate.

$$\frac{\sigma(Y_{2500}(R_0 + R_1))}{\sigma(Y_{2500}R)}$$

Across plates, the mean swapping fraction is 2.653 $\pm$ 0.444% (Supplementary Figure 18).

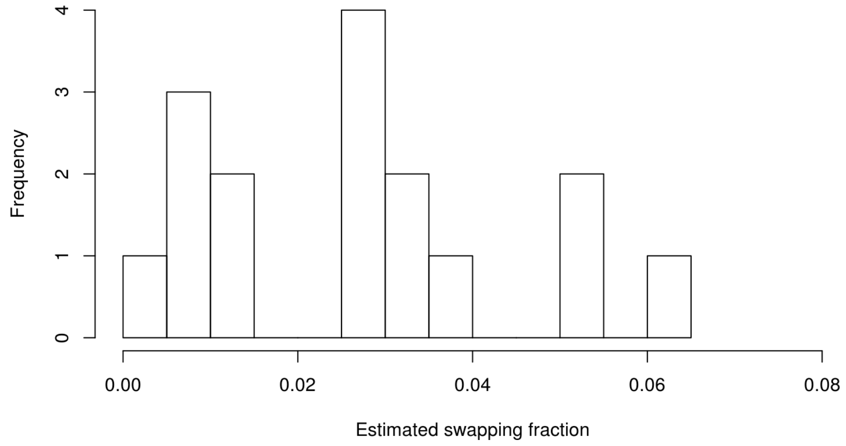

Supplementary Figure 18: Estimates of the swapping fraction for all plates, using the top 500 most informative genes for each plate.

We obtained similar results with the top 250 (2.581 $\pm$ 0.4%) or 1000 (2.707 $\pm$ 0.503%) most informative genes per plate. We also repeated the analysis with a random selection of 500 genes from the top 1000 most informative genes per plate, which yielded similar estimates (2.977 $\pm$ 0.539%). The variance of the swapping fraction estimates for different numbers of informative genes on the same plate is smaller than the variance across plates (Supplementary Figure 19), indicating that the number of informative genes used in the model fit is not the major contributor to differences in the estimates between plates.

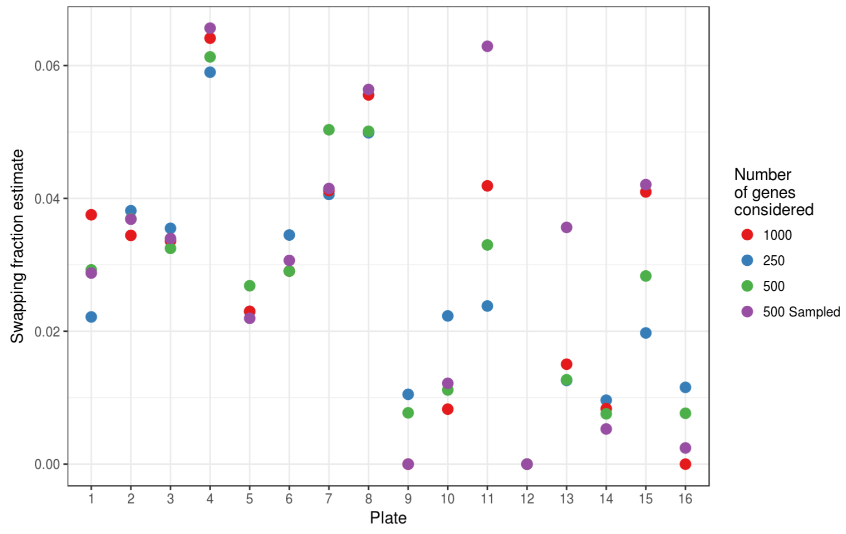

Supplementary Figure 19: Swapping fraction estimates for all plates, using different numbers of informative genes in the model fit. '500 Sampled' refers to 500 genes selected at random from the top 1000 genes.

In the Richard dataset, we considered swapping along rows and columns exclusively. We use a similar approach here by considering the fraction

$$\frac{\sigma(Y_{2500}R_1)}{\sigma(Y_{2500}R)}$$

This yields an estimated row-column swapping fraction of 2.068 $\pm$ 0.326%. These row-column swapping fractions are well-correlated with the total swapping fractions (Supplementary Figure 20).

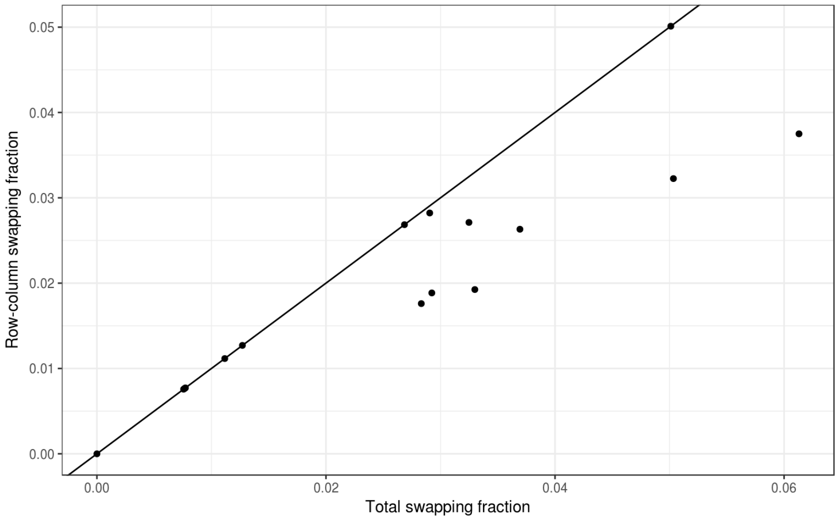

Supplementary Figure 20: Relationship between the total swapping fraction and the row-column swapping fraction. The identity line is shown for comparison.

The mean value of the information score for the top 500 genes (excluding `Inf`) is not associated with the estimated swapping fraction (Supplementary Figure 21). This indicates that differences in the availability of genes with high information score do not drive differences in the swapping fraction estimates.

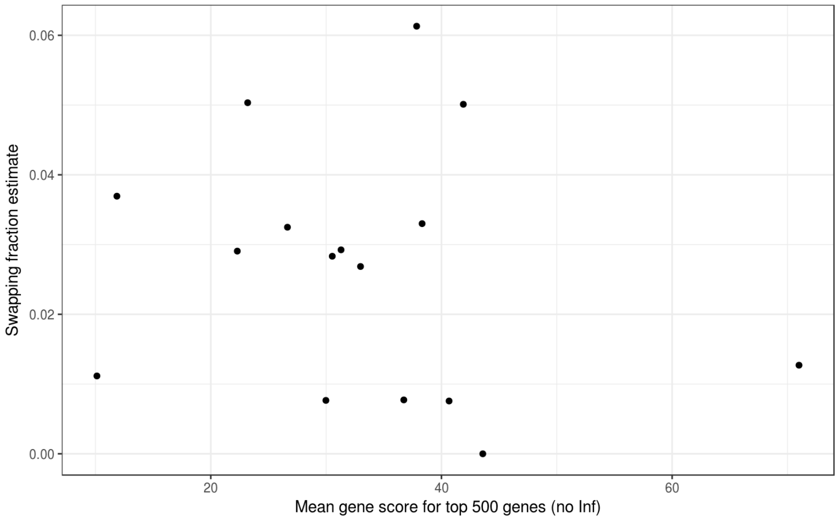

Supplementary Figure 21: Relationship between the swapping fraction estimate from each plate and the mean information score for the top 500 genes.

Finally, recall our initial assumption that the swapping fraction on the HiSeq 2500 is negligible compared to that on the HiSeq 4000. However, some swapping does occur on the HiSeq 2500, at approximately one tenth the rate as the HiSeq 4000 (as shown in the Richard data). Thus, our estimate of the swapping fraction actually represents the relative increase in swapping in the HiSeq 4000 compared to the HiSeq 2500. We can approximate the absolute swapping fraction in the HiSeq 4000 by multiplying our estimate by 1.1. This

yields an swapping fraction estimate of 2.275 $\pm$ 0.359% along rows and columns, which is very similar to the value calculated in the Richard data (2.180 $\pm$ 0.0765%).

Linking swapping fraction to library characteristics

In an attempt to understand the variance of our swapping fraction estimates, we examined the concentration of free barcodes on each plate. Using the Bioanalyzer Expert software (Supplementary Figure 22), we quantified the barcode concentration in each multiplexed pool based on the area under the peak at 40-75 bp. By comparison, sequenced cDNA should fall within the peak at 400-800 bp.

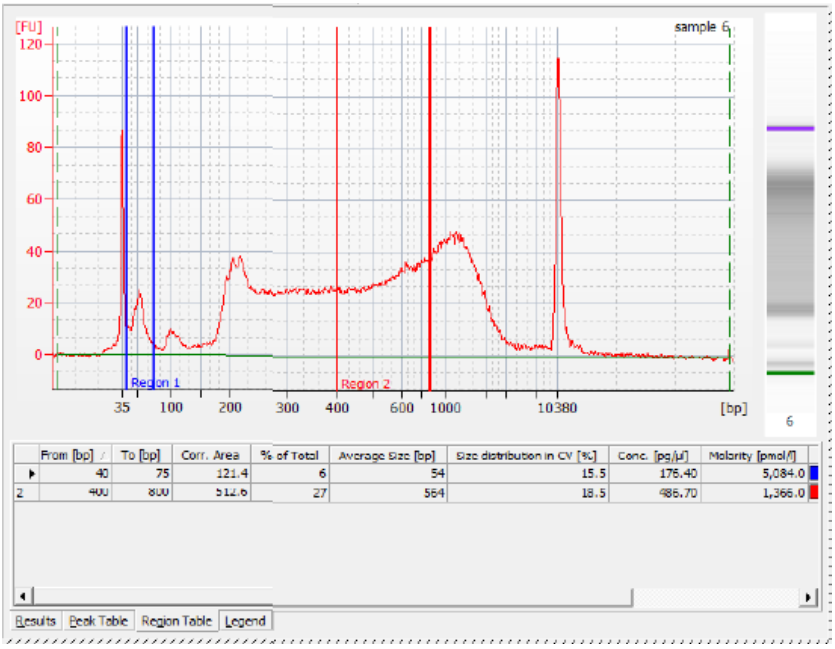

Supplementary Figure 22: Screenshot of an analysis of molecule lengths from a single plate, using the Bioanalyzer Expert software. Region 1 (40-75 bp) corresponds to free DNA barcode, while region 2 (400-800 bp) corresponds to cDNA that can be sequenced on the HiSeq 4000.

The barcode and cDNA molarities for all samples are shown in Supplementary Figure 23, overlaid with the calculated swapping fractions.

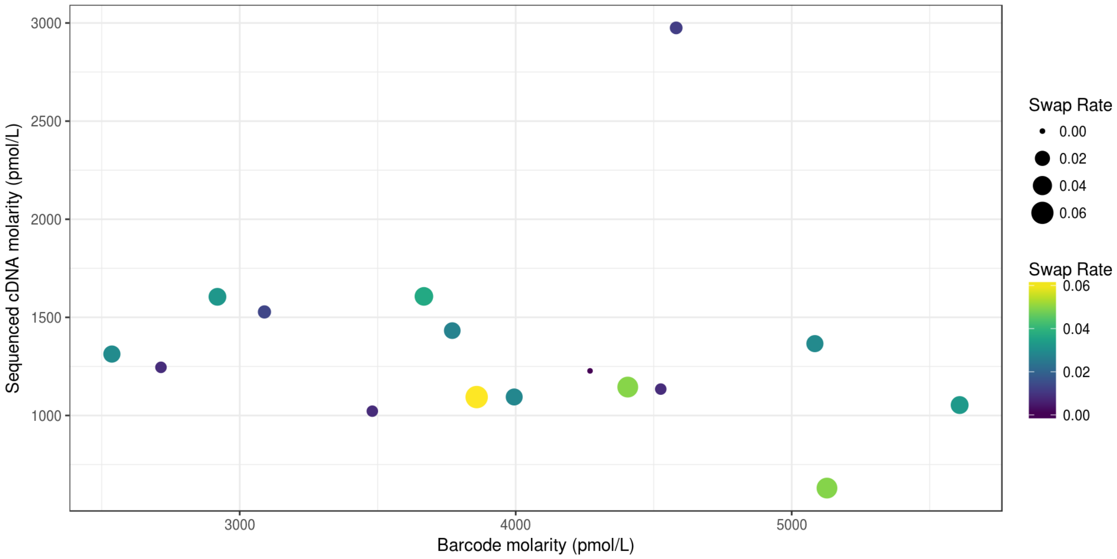

Supplementary Figure 23: Molarities of cDNA and free barcode for each plate, quantified using the Bioanalyzer software. The size and colour of the point corresponding to each plate is determined based on its estimate of the swapping fraction.

We did not observe any obvious association between these measures. This is demonstrated more clearly in Supplementary Figure 24, where the swapping fractions are plotted directly against the molarity of free barcode.

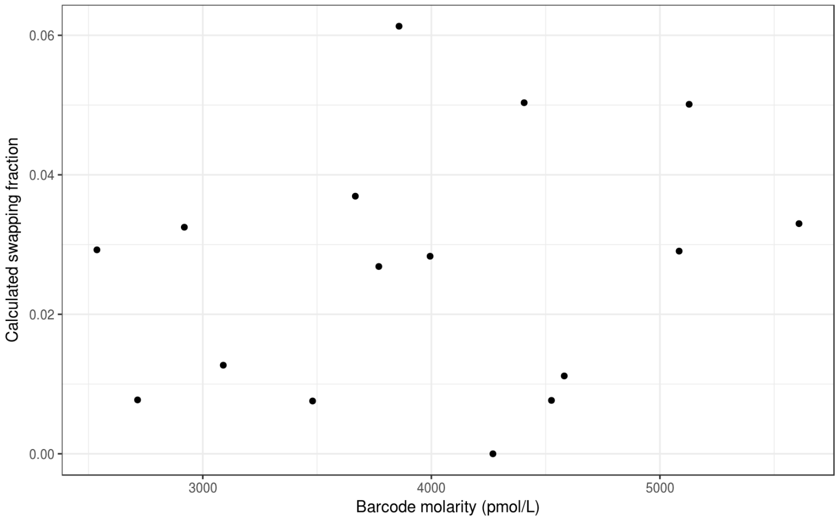

Supplementary Figure 24: Estimated swapping fraction for each plate, plotted against the molarity of free barcode.

The gradient in a linear model fitted to the swapping fraction against the barcode molarity is not significantly different from 0 (p=0.427). Similarly, we do not observe any correlation between the swapping fraction and the ratio of free barcode to captured cDNA (Supplementary Figure 25).

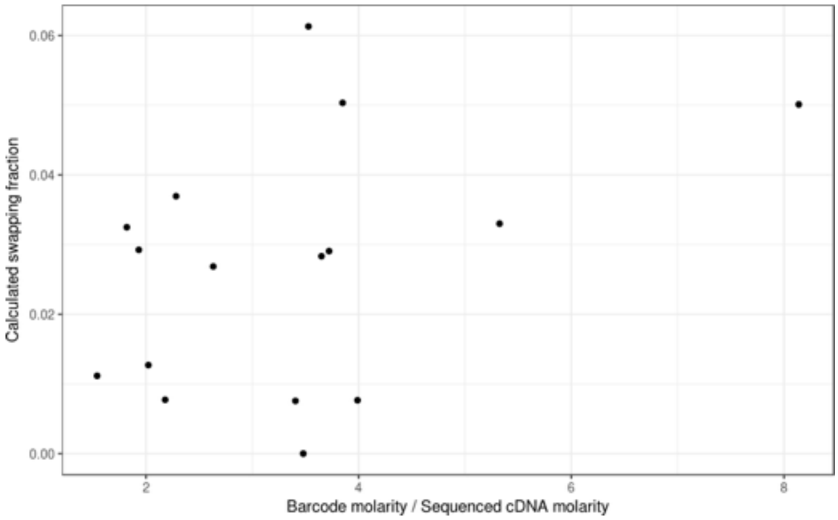

Supplementary Figure 25: Estimated swapping fraction for each plate, plotted against the ratio of free barcode concentration to cDNA concentration.

Again, the slope in the fitted linear model is not significantly different from 0 (p=0.129, p=0.466 after removing the high-ratio outlier at the right).

The molarity calculations for the sequenced cDNA may contain barcode concatamers, which do not align to the mouse genome and are irrelevant to our swapping fraction estimation. These concatemers may distort the estimate for the concentration of cDNA on each plate. To overcome this, we used the total number of mapped reads per plate as a proxy for the amount of sequenced cDNA (Supplementary Figure 26), and examined its relationship with the swapping fraction.

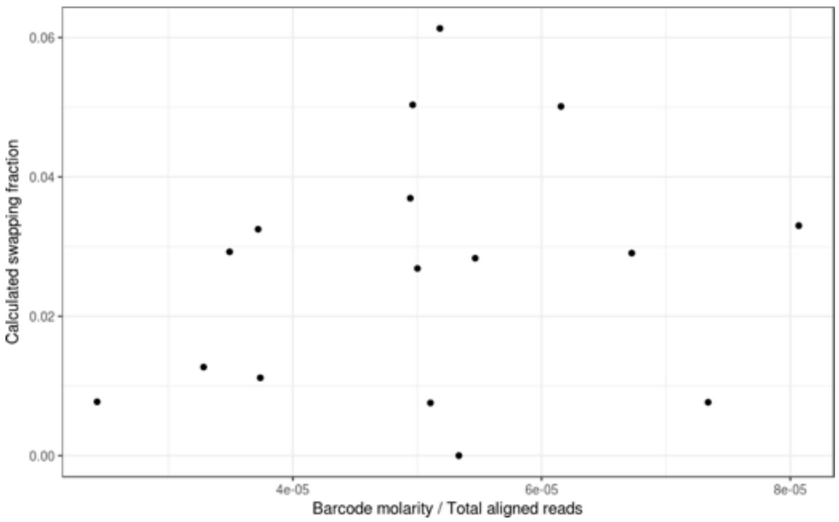

Supplementary Figure 26: Estimated swapping fraction for each plate, plotted against the ratio of free barcode concentration to the total number of mapped reads.

However, we still did not see a slope significantly different from 0 (p=0.452).

In summary, our calculated swapping fractions are not associated with the amount of free barcode in libraries, nor the ratio of free barcode to cDNA concentration. This contrasts with Sinha et al. (2017), who showed that titrations of increasing amounts of additional free primer (from 1 to 100 nM) increased the rate of barcode swapping. We note that their clearest result used 100nM of free barcode, which is far in excess of standard experimental quantities. Our analysis suggests that the concentration of free barcode has little bearing on the rate of swapping at typical experimental levels (2-6 nM in this data).

Testing for transcriptome-wide swapping

In Supplementary Figures 11 and 12, we showed a crosshair swapping pattern for a single gene for a single plate. We now apply a model to each gene across all plates to determine whether swapping is happening consistently across genes. This differs from the previous model, which used information from many genes simultaneously to obtain a global estimate of the swapping fraction for each plate.

Let i denote the row index for a cell on a single 96 well plate ($1 \leq i \leq 8$) and let j denote a cell's column index ($1 \leq j \leq 12$). Let $Y^{(t)}_{i,j,g}$ denote the read count of gene g in cell (i, j) when profiled using sequencing technology t, where $t = 2500$ refers to the HiSeq 2500 and $t = 4000$ refers to the HiSeq 4000. Additionally, let

$$S^{(t)}_{i,j,g} = \sum_{k=1}^{12} Y^{(t)}_{i,k,g} + \sum_{l=1}^{8} Y^{(t)}_{l,j,g} - 2Y^{(t)}_{i,j,g}$$

represent the total read count of gene g in technology t across all other cells on this plate sharing an index with (i, j).

We first consider a null model without swapping where, for cell (i, j), the number of reads mapped to each gene g in the HiSeq 4000 dataset is only dependent on the number of reads mapped to the same cell in the HiSeq 2500 data. This means that

$$H_0 : Y^{4000}_{i,j,g} = \alpha_g Y^{2500}_{i,j,g} + \epsilon_{i,j,g}$$

α_g captures both read depth differences as well as any gene specific biases (e.g., GC content) that may change between the two sequencing machines. $\epsilon_{i,j,g}$ represents the residual error.

We also consider an alternative model with a swapping term, where each cell in the HiSeq 4000 data receives swapped reads from (or transfers swapped reads to) cells that share exactly one barcode:

$$H_1 : Y^{4000}_{i,j,g} = \alpha_g Y^{2500}_{i,j,g} + \beta_g \left(S^{2500}_{i,j,g} - Y^{2500}_{i,j,g} \right) + \epsilon_{i,j,g}$$

β_g allows the strength of the swapping term to vary between genes, so that we can determine whether all genes are consistently affected by swapping. $S^{2500}_{i,j,g}$ represents the pool of available reads from donor libraries that can be transferred into the recipient library for cell (i, j) due to swapping of one barcode. $Y^{2500}_{i,j,g}$ represents the pool of reads that can be transferred from the donor cell (i, j) to other recipient libraries. (This represents the "loss" of PCR duplicate reads due to barcode swapping.) $\epsilon_{i,j,g}$ represents the residual error.

Models H_1 and H_0 are fitted by least squares to high-abundance genes, i.e., mean counts greater than 50 across all plates. This focuses on genes that have sufficient reads for swapping to clearly manifest itself. In contrast, genes with low or zero counts provide no information about the presence or absence of swapping. The use of least squares assumes a normal distribution for the errors, which is reasonable for large Poisson-distributed counts.

For each gene, evidence against H_0 in favour of H_1 is established by performing an F-test (as the models are nested). The use of the swapping term significantly improves the model fit to the data when H_1 is favoured over H_0. In addition, we expect that $\beta > 0$, i.e., swapping transfers reads from donor libraries to recipient libraries. If $\beta < 0$, the behaviour of swapping is inverted compared to the other work presented above.

When the model was applied to the Nestorowa dataset, the vast majority of genes favoured H_1 with $\beta > 0$, as shown in Supplementary Figure 27.

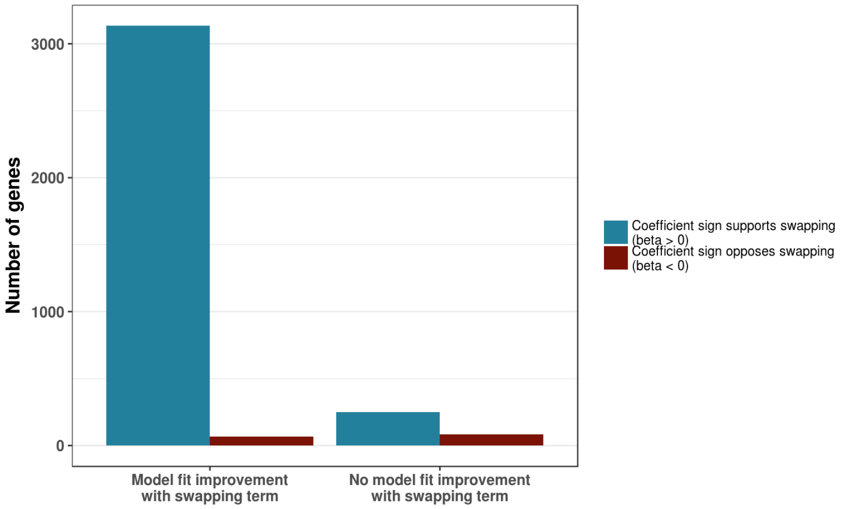

Supplementary Figure 27: Number of genes where the alternative swapping model (H_1) offers a significantly improved fit over the null model (H_0) with a positive (blue) or negative estimate of β_g (red).

Of all tested genes, 90.5% exhibited a significant improvement in the model fit with the swapping term (adj. $p < 0.05$). Of these significant genes, 97.9% have a positive value of β, supporting the expected barcode swapping model.

This strongly suggests that barcode swapping is more prevalent on the HiSeq 4000 compared to the HiSeq 2500, and that it affects nearly all tested genes.

As a negative control, we also fitted these models to an experiment sequenced only on the HiSeq 2500. Specifically, we considered the same model as described above, but replacing the HiSeq 4000 counts with a replicate sequencing run of the same multiplexed pool that was also sequenced on the HiSeq 2500. Here, only 0.099% of genes have a significantly improved fit with the β term, as there is no increased incidence of swapping between the two lanes, supporting the robustness of the model used.

Supplementary Note 5: Droplet-based analyses

Introduction

Recently developed single-cell RNAseq protocols use microfluidic systems to automate stages of library preparation by capturing individual cells in droplets. Each run of the microfluidic system generates a sample that typically contains thousands of cells. These droplet-based protocols label their cells in a different manner to plate-based assays, as a cell barcode unique to each droplet is incorporated into the transcript alongside an additional Illumina barcode that labels different sets of cells (i.e., each set of cells is a single sample in droplet-based experiments). Swapping of Illumina barcodes will move transcripts between samples while retaining the same cell identifier (Supplementary Figure 28).

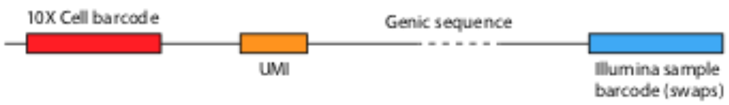

Supplementary Figure 28: Schematic of the barcoding strategy used in 10X Genomics experiments. Each captured cDNA contains multiple barcodes, including a 10X-supplied cell-labelling barcode, a randomly generated unique molecular identifier (UMI), and an Illumina-supplied sample index. Only the sample index is expected to swap, leaving the cell barcode and UMI unchanged.

As discussed in the main text, swapping of the sample barcode can have two effects depending on whether the same cell barcodes are present in both the donor and recipient samples. If they are, the expression profile for each shared cell barcode in the recipient sample will become a mixture with the corresponding profile in the donor sample. This is equivalent to the effect in plate-based assays, as discussed above for the Richard and Nestorowa datasets. Otherwise, artefactual cells may appear in the recipient sample, corresponding to the swapped-in cell barcodes from the donor sample. This manifests as an increase in the number of shared cell barcodes between samples.

Barcode swapping is additionally problematic with UMI data where multiple reads for the same captured cDNA molecule are collapsed into a single UMI count. Even a very small number of swapped reads for a molecule will constitute a single count in the recipient sample. This means that the

contribution of a few swapped reads will be the same as the contribution of a molecule that is sequenced hundreds or thousands of times in its sample of origin. In this manner, the effect of barcode swapping on the recipient expression profile is effectively inflated in UMI data compared to read count data.

Shared cell barcodes between multiplexed samples

Description of the null model

Here, we investigate whether swapping of the sample barcode causes an excess of cell barcode sharing between multiplexed droplet-based scRNA-seq samples. Assuming that cell barcodes are drawn at random for each sample, we can formulate a null model for the proportion of shared cell barcodes between two samples.

We used data generated from the 10X Chromium system, where there are "approximately 750,000" unique cell barcodes (Zheng et al. 2017). Specifically, utilisation of CellRanger returns a raw count matrix of 737,280 columns as output for each sample. This does not include any cell filtering. We therefore consider this value as the total number of cell barcodes N. For samples 1 and 2, we have n_1 and n_2 cell barcodes, respectively, that have been called as cells. We further denote the number of shared cell barcodes observed between samples 1 and 2 as S.

Under the null hypothesis (i.e., no barcode swapping between samples), cell barcodes are drawn independently in each sample. In this case, the number of shared cell barcodes between samples should follow a hypergeometric distribution. This is based on randomly drawing n_2 cell barcodes from the total set of N cell barcodes without replacement. The number of successes is defined as the number of drawn cell barcodes that are also in the set of n_1 cell barcodes called in sample 1.

We use this distribution to compute a p-value for the observed S, i.e., how often would we expect to see a result as or more extreme ($\geq S$) given random drawing of cell barcodes from the pool? This is repeated for every pair of samples to examine all possible swapping relationships.

Dataset 1 on the HiSeq 2500

Here, droplet data was generated from mouse embryonic cells using the 10X Genomics Chromium system and sequenced using a HiSeq 2500 machine. 11 samples were multiplexed, varying in size between 3776 and 313 called cells. CellRanger 1.3.1 was used for sample processing with default arguments. Supplementary Figure 29 shows p-values from the hypergeometric tests between every pair of samples.

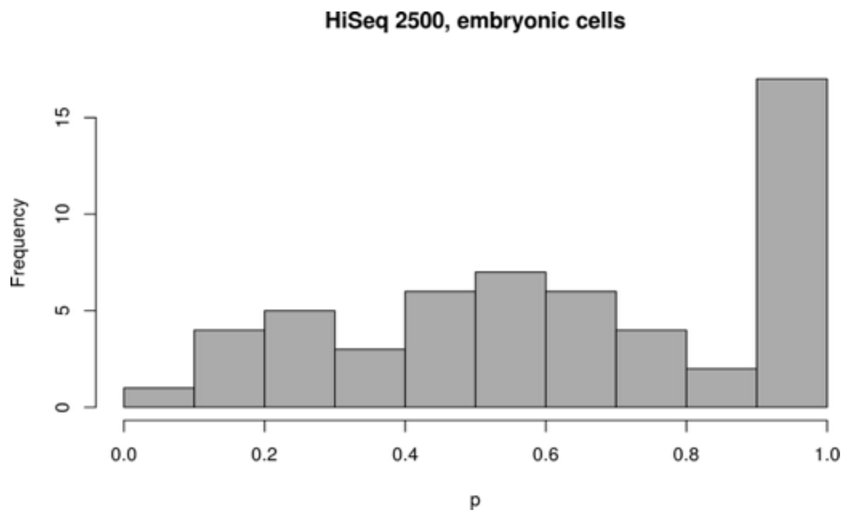

Supplementary Figure 29: Histogram of p-values for cell barcode sharing between all pairs of 10X samples in dataset 1, sequenced on the HiSeq 2500.

None of the p-values for any comparison were significant after FDR correction at any significance level (all adj. p-values equal to 1). Thus, we do not observe any excess of sharing in this data, consistent with the low swapping fraction on the HiSeq 2500.

Dataset 2 on the HiSeq 2500

We repeated our analysis on another dataset involving mouse epithelial cells, processed using the 10X Chromium system and sequenced on a HiSeq 2500. CellRanger 1.2.1 was used for data processing with default arguments. The six samples range in size between 1102 and 135 called cells. Two samples were excluded from analysis here due to failed library preparation. Only 2 cell barcodes were shared between samples, so p-values are almost universally 1 (Supplementary Figure 30). Again, there is no excess of barcode sharing.

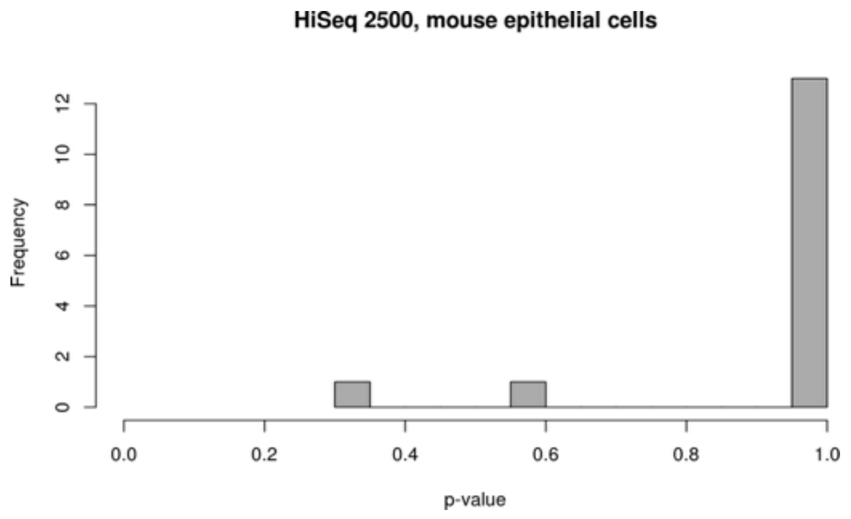

Supplementary Figure 30: Histogram of p-values for cell barcode sharing between all pairs of 10X samples in dataset 2, sequenced on the HiSeq 2500.

Dataset 2 on the HiSeq 4000

The same mouse epithelial cell dataset (Dataset 2, above) was also sequenced on the HiSeq 4000. In this data, the six samples range in size between 1111 and 151 called cells. Two samples were again excluded from analysis here due to failed library preparation. Here we observe low p-values in all pairwise comparisons (Supplementary Figure 31), consistent with increased barcode swapping on the HiSeq 4000.

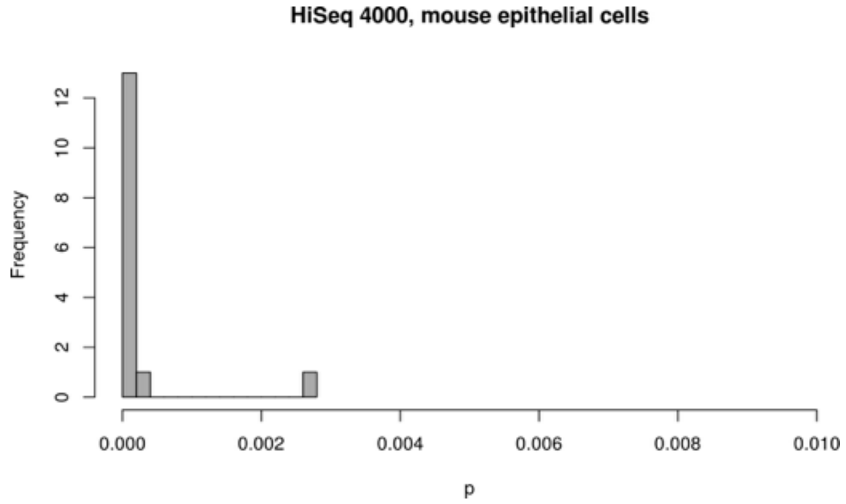

Supplementary Figure 31: Histogram of p-values for cell barcode sharing between all pairs of 10X samples in dataset 2, sequenced on the HiSeq 4000. Note that the x-axis scale differs from the previous histograms.

Despite this, the absolute rate of cell barcode sharing is still low. Of 2950 barcodes, only 10 cell barcodes occur more than once across all samples in this dataset.

Dataset 3 on the HiSeq 4000

This dataset contains four samples of human xenograft cells, generated using the 10X Genomics Chromium system. Libraries were sequenced on a HiSeq 4000, and data was processed using CellRanger 1.3.1 using default arguments. Samples varied in size between 4608 and 1462 called cells. Of the 9621 cell barcodes, 16 were observed twice, with none observed three times or more.

Supplementary Figure 32 shows p-values from the hypergeometric tests between every pair of samples. One pairwise comparison contains a significant excess of sharing after FDR correction (adj. p-value of 0.0594). In this comparison, 9 cell barcodes were shared compared to an expected value of 3.45 for samples of size 1771 and 1462.

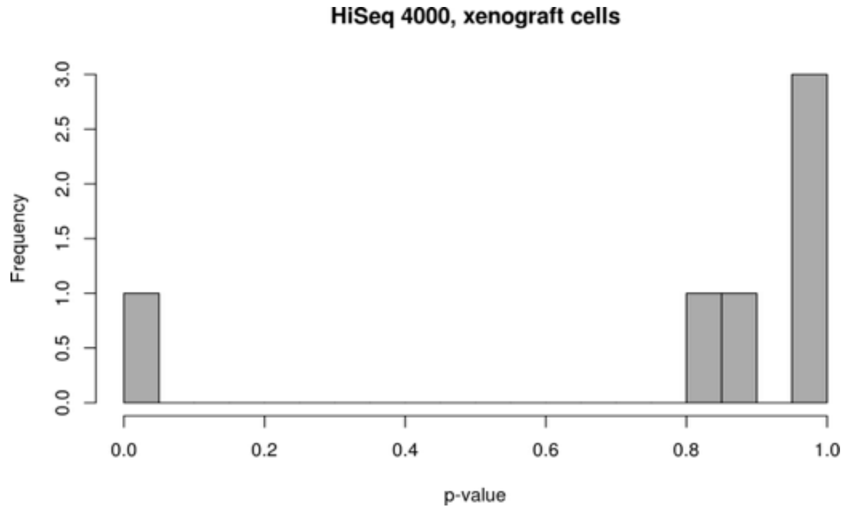

Supplementary Figure 32: Histogram of p-values for cell barcode sharing between all pairs of 10X samples in dataset 3, sequenced on the HiSeq 4000.

In both HiSeq 4000 datasets, we observe excess sharing of cell barcodes between samples. However, the actual number of shared cell barcodes remains low. We hypothesise that, due to the low rate of barcode swapping,

swapped-in cell libraries (i.e., the potentially artefactual libraries) in the recipient samples are very small compared to the libraries of real cells. As a result, the swapped-in libraries are mostly discarded by cell-calling algorithms that distinguish cell-containing and empty droplets based on their library sizes. This suggests that droplet data has an intrinsic robustness to the generation of artefactual cells, at least when samples are of high quality and contain cells of comparable size.

Artefactual cells appear due to barcode swapping in compromised samples

In one experiment, we sequenced the transcriptomes of cells from four different experimental conditions (A-D), using two biological replicates for each condition (1-2).

Supplementary Figure 33 shows a t-SNE plot for the cell transcriptomes after application of a typical scRNA-seq analysis pipeline. Clusters were identified using the Louvain clustering algorithm on a shared nearest neighbour graph (Xu and Su 2015) considering the 10 nearest neighbours per cell.

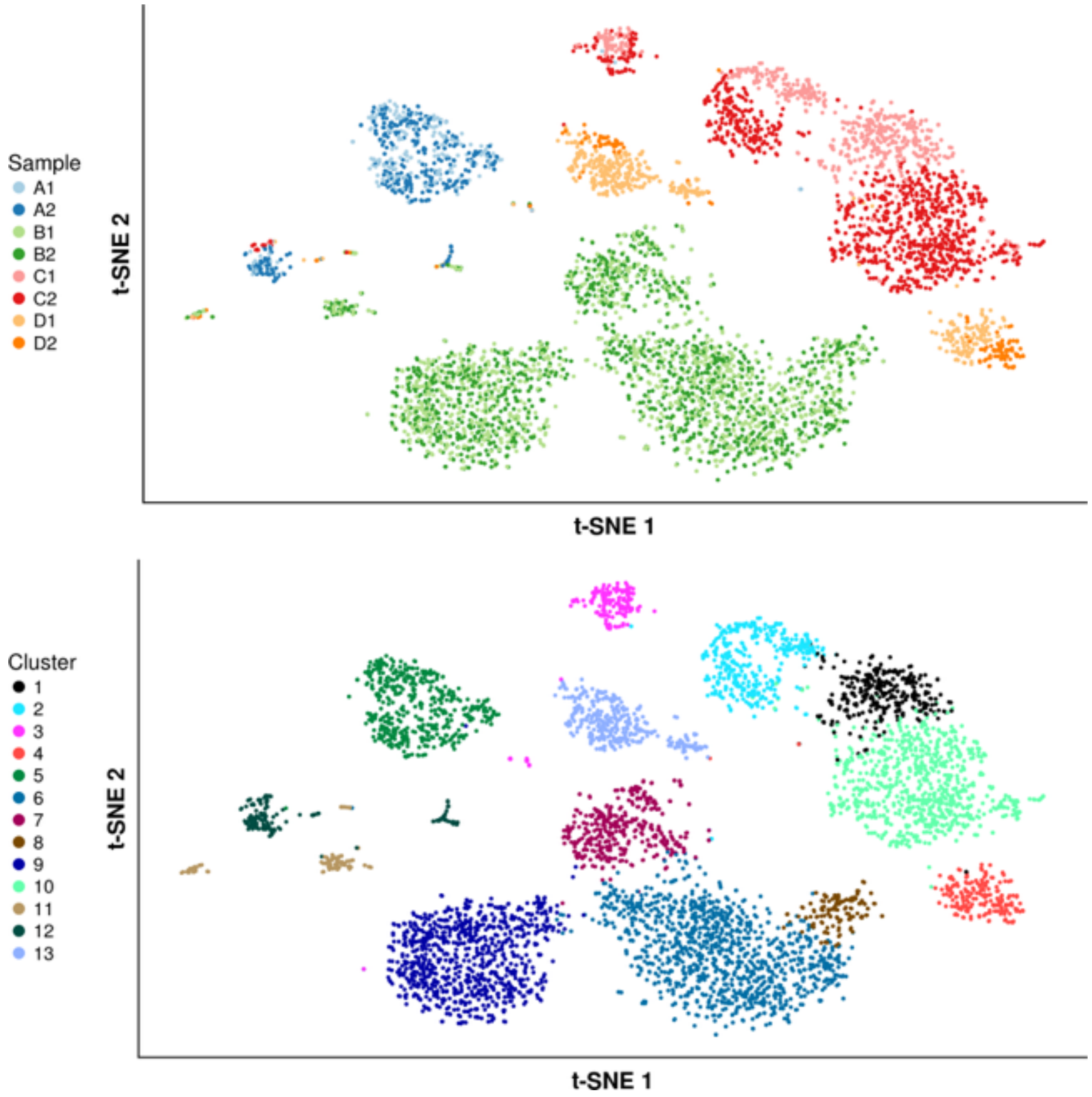

Supplementary Figure 33: t-SNE of cell transcriptomes in a 10X experiment sequenced on the HiSeq 4000. Each point represents a cell and is coloured by sample (top) or cluster (bottom).

This appears to show unique populations of cells for each experimental condition. Upon closer inspection, we found that samples B1 and B2 contained cells with considerably smaller library sizes (Supplementary Figure 34). Importantly, we observed that B1 and B2 shared almost all of their cell barcodes with each other and with other samples that were multiplexed with them (Supplementary Figure 35). This behaviour is clearly unusual. (Accordingly, samples B1 and B2 were excluded from the earlier analyses in Supplementary Figures 30 and 31.)

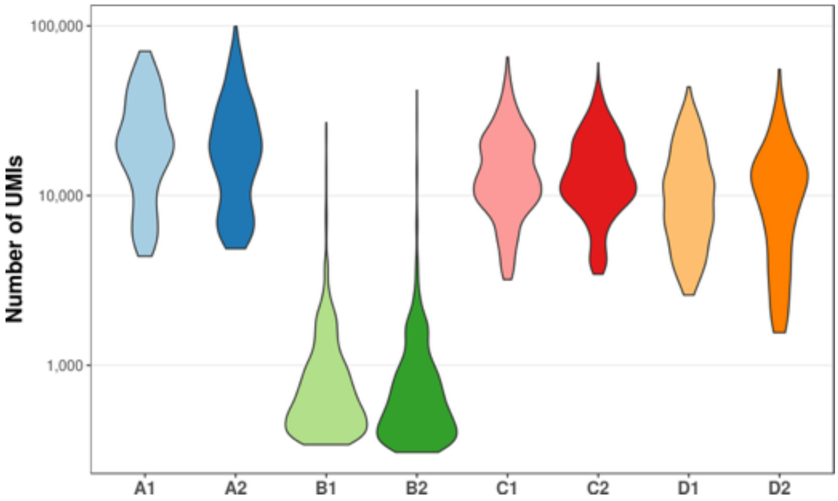

Supplementary Figure 34: Distribution of UMI count across all cell barcodes in each 10X sample. Only cell barcodes that were detected by CellRanger as cell-containing droplets are shown.

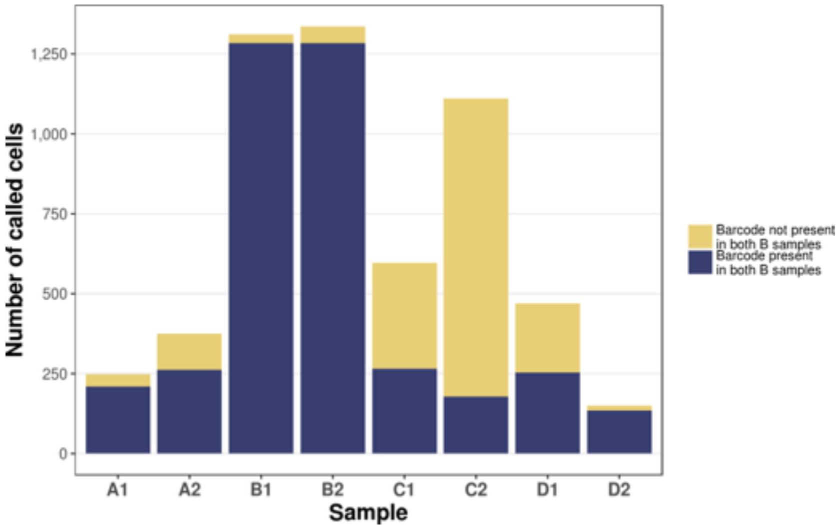

Supplementary Figure 35: Number of cell barcodes in each sample that are also present in both samples B1 and B2. The number of cell barcodes in each sample that are not present in both B1 and B2 is also shown.

We hypothesized that the cells in samples B1 and B2 were damaged prior to or during library preparation in the 10X Chromium system. This resulted in the very small library sizes observed for cells in these samples, as the actual amount of input RNA in each droplet was very low. Because the real cell libraries in the B samples were very small, barcode swapping from other samples generated artefactual libraries that were large relative to the real cells. These swapped libraries were subsequently called as cells by the CellRanger software, despite being derived purely from other samples. This is supported by the excessive sharing of barcodes between the two B samples and with the other high-quality samples.

Why have these small libraries been called as cells? This is due to CellRanger's cell calling algorithm, which defines cells as all barcodes with a total UMI count greater than or equal to 10% of the 99th percentile of the expected number of recovered cells (Zheng et al. 2017) (this is the case as of Cellranger 2.1.0, the latest version at time of writing). If the 99th percentile is low, many barcodes with low library sizes will be called as cells, regardless of whether the corresponding libraries are noisy, of poor quality, or derived from swapping. This is illustrated in Supplementary Figure 36, where the rapid drop-off in library size for sample B1 results in a smaller value for the 99th percentile (assuming that we expect to obtain around 2000 cells) compared to another sample.

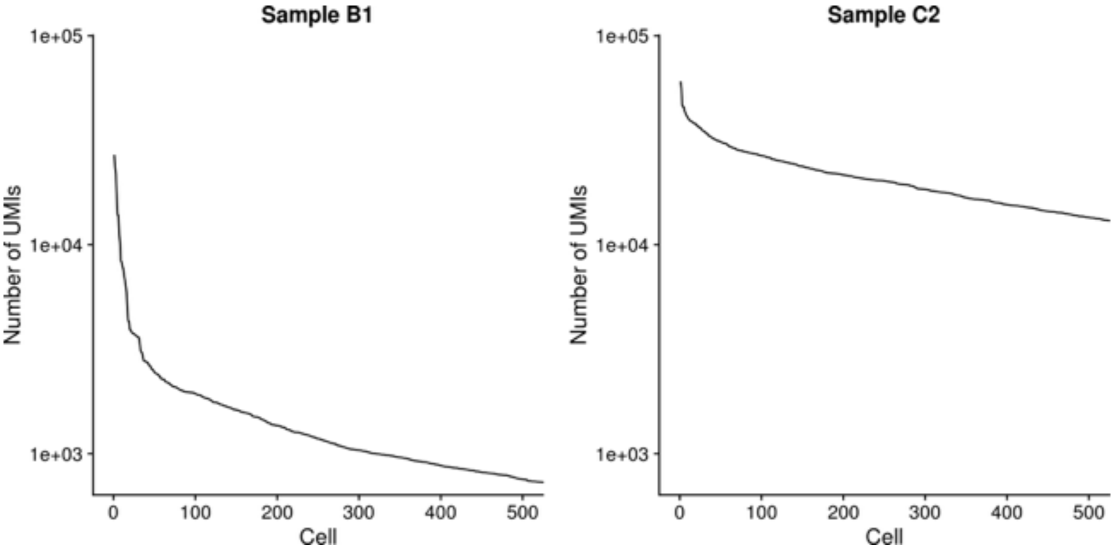

Supplementary Figure 36: Total number of UMIs in each cell barcode for samples B1 and C2, in decreasing order for the top 500 barcodes.

Finally, not all barcodes from high-quality samples are observed in the poor quality B samples. What may drive a barcode's presence or absence? Consider differences in library size: a large barcode library has more total cDNA in the sequencer, and will therefore contribute more swapped reads to other samples. Conversely, a smaller library will contribute fewer swapped reads. Therefore, when calling cells based on size, we would be more likely to call an artefactual cell generated by swapping from a large library.

Based on this reasoning, barcodes from high-quality samples that are also called as cells in the B samples should have larger libraries than other barcodes that are present in only the high-quality samples. Indeed, Supplementary Figure 37 demonstrates that shared-barcode libraries do have significantly more molecules ($p = 9.96e-259$, with a ratio of 2.2 between the medians). This confirms that the cells called in B are largely derived from swapping artefacts.

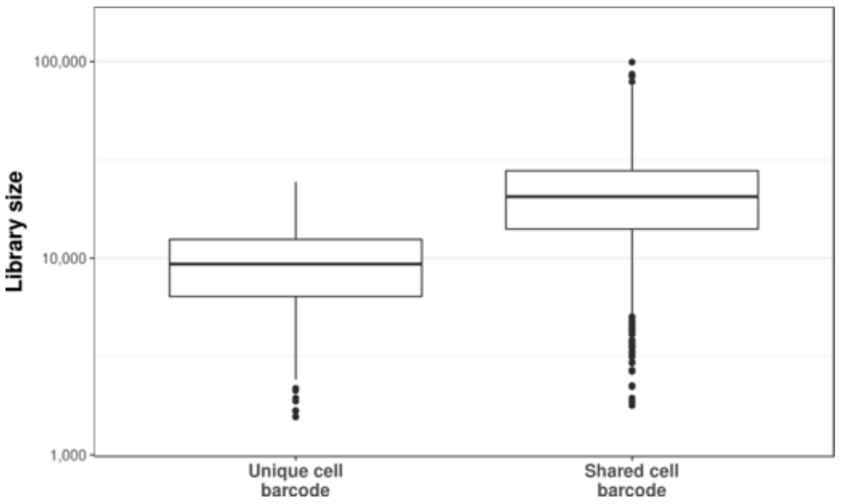

Supplementary Figure 37: Library sizes of cell barcodes in high-quality samples that are shared with B1/B2 (“Shared cell barcode”) or not (“Unique cell barcode”). Dots represent cell barcodes that are more than 1.5 interquartile ranges from the edges of each box.

Given this, one might expect the swapped libraries to cluster with the cell populations from which these libraries truly derived. However, this is clearly not the case. We hypothesise that this is due to the presence of cell types specific to samples B1 and B2 that are lysed during sample preparation. Their RNA is subsequently released into solution and captured in cell-free droplets that share a cell barcode with the swapped artefactual cells from other samples. The expression of one such gene is shown in Supplementary Figure 38.

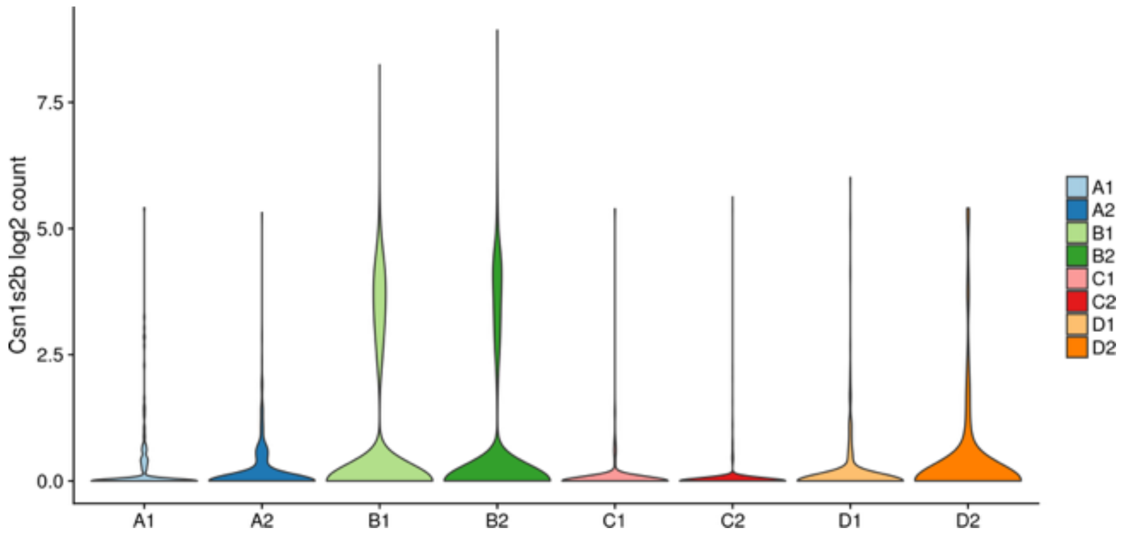

Supplementary Figure 38: Violin plot of gene expression in the called cells from each sample.

To confirm that barcode swapping was driving this behaviour, we resequenced the libraries on HiSeq 2500. This resulted in the loss of many called cells from samples B1 and B2 (Supplementary Figure 39), consistent with the generation of artefactual cells due to swapping on the HiSeq 4000. By comparison, cell numbers for the other samples are largely unaffected.

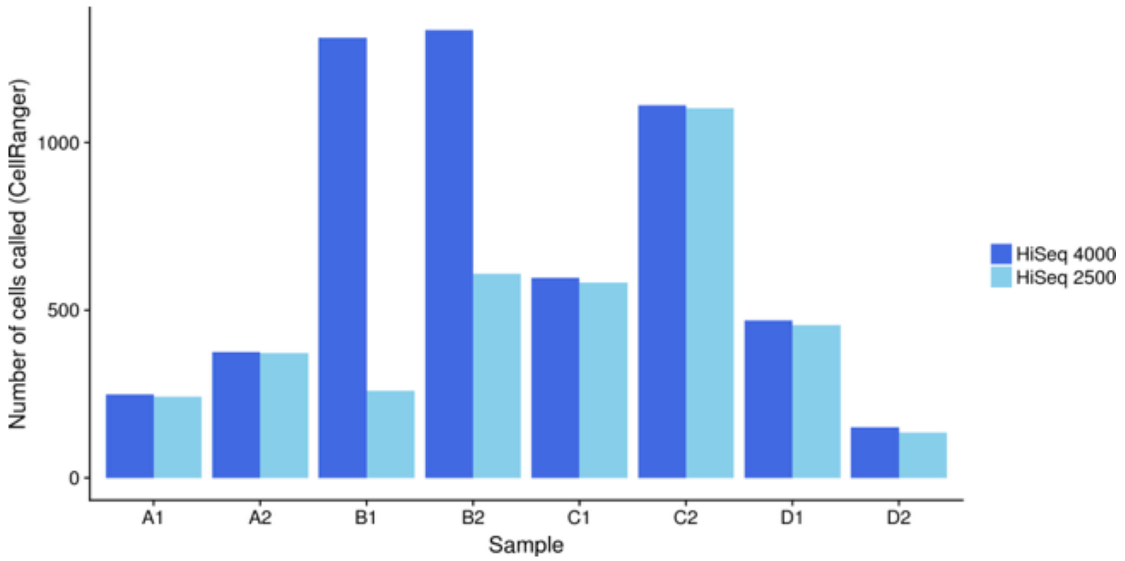

Supplementary Figure 39: Number of called cells in each sample in the HiSeq 4000 dataset and in the resequenced HiSeq 2500 dataset.

A t-SNE of the transcriptomes in the HiSeq 2500 dataset is shown in Supplementary Figure 40. We note that the number of cells remaining in samples B1 and B2 is comparable to that in the other samples. This is likely due to the previously described problems with the 99th percentile calling strategy.

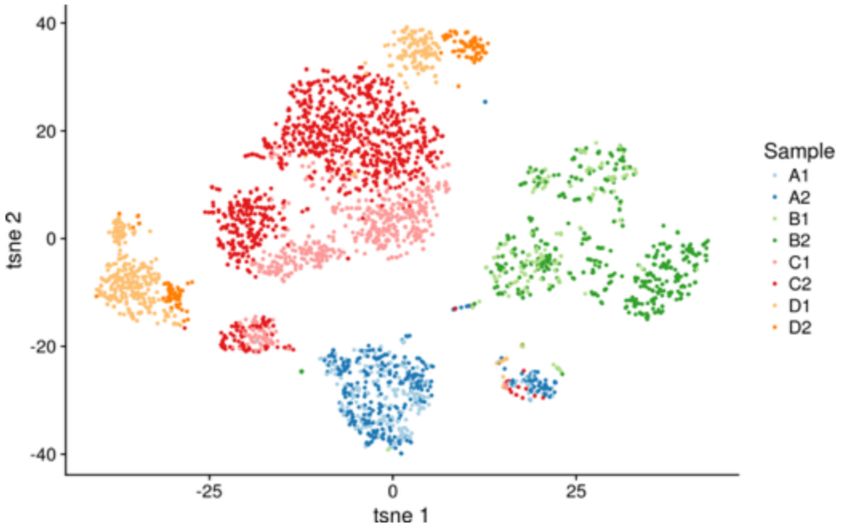

Supplementary Figure 40: t-SNE of cell transcriptomes in a 10X experiment resequenced on the HiSeq 2500. Each point represents a cell and is coloured by sample.

This dataset is a particularly egregious example of the misleading effects of barcode swapping. Without an inspection of barcode sharing between samples, we might have concluded that a separate cell population was present in samples B1 and B2 (Supplementary Figure 33). This would be incorrect as there are, in fact, very few genuine cells in those samples. Even in more typical datasets, similar problems may arise due to differences in capture efficiency or RNA content between cells. Swapping from a high-cDNA library in a donor sample will generate an artefactual library in a recipient sample that is indistinguishable from a low-cDNA library generated from a real cell. This makes it difficult to be certain that cells actually exist in their labelled sample.

Supplementary Note 6: Removing barcode-

Cell exclusion

We recommend testing for the degree of cell barcode sharing between samples as a standard quality control step in droplet-based single-cell experiments. The method we have presented above (a hypergeometric test on pairwise comparisons) is quick and easy to apply.

If problems with barcode swapping are observed, the easiest solution is to **remove any cells labelled with a barcode that exists in more than one sample in each multiplexed sequencing run**. This procedure will remove the artefacts, regardless of whether the cells truly exist in both samples (which will cause transcriptome mixing) or whether artefactual cells are being created. The cost of such a procedure will be the loss of some genuine cells for downstream analysis, even in the absence of swapping.

We have run simulations to understand how much data are discarded by excluding shared cell barcodes, even in a perfect experiment (i.e., without any swapping). We consider a simulated experiment where 8 samples are multiplexed together. Each sample consists of an equal number of cells n, whose barcodes are drawn at random with replacement from 10X's set of 737,280. Barcodes are drawn with replacement, as the pool of 10X gel beads does not contain exactly one of each barcode. Drawing of barcodes is independent between samples, assuming no creation of artefactual cells due to swapping. Barcodes are then marked for exclusion if they are observed in more than one of the eight samples. For each n, we consider the mean of 100 simulations.

Supplementary Figure 41 shows the rate of cell removal for different values of n, and different numbers of multiplexed samples. The expected rate of removal is low (< 5%) at low n with few samples, corresponding to a small experiment. In such cases, cell exclusion is a simple and effective strategy for removing swapping artifacts without discarding much data. However, it is not suitable for datasets with many $(n > 10000)$ cells per sample, where the expected rate of removal rapidly increases. Similarly, it should not be used in cases where many samples are multiplexed for sequencing, which will also increase the exclusion rate and lead to unnecessary loss of data.

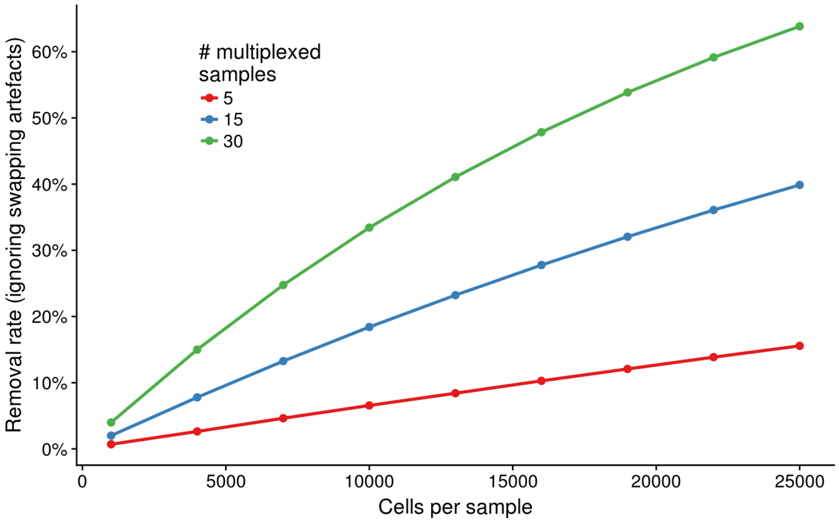

Supplementary Figure 41: Percentage of cell barcodes that are incorrectly discarded by a cell exclusion approach, irrespective of barcode swapping. This is based on simulated data for an experiment containing either 5, 15, or 30 multiplexed 10X samples each containing the same number of cells. The real exclusion rate is likely to be larger than these values, as artefactual cells due to barcode swapping are not considered here.

Molecule exclusion

Background

A more precise solution for removing swapping effects focuses on the cDNA molecules themselves. In a 10X Genomics single-cell experiment, transcripts are generated that contain:

- An Illumina sample barcode
- A cell barcode, drawn from a pool of 737,280
- A unique molecular identifier (UMI): a 10 bp random sequence, providing 1,048,576 combinations
- Gene sequence from the captured mRNA

The chance of observing a read in two different samples with the combination of same cell barcode, UMI, and gene alignment is extremely low, due to the large amount of combinatorial complexity. We therefore assume that all reads with the same combination are derived from a single original molecule.

We first considered the mouse epithelial cells sequenced on the HiSeq 4000 (i.e., dataset 2). We identified reads with the same combination of UMI, cell barcode and gene across all samples. For each combination, we calculated the fraction of all reads that were observed in each sample, and obtained the largest read fraction across all samples. Supplementary Figure 42 shows the distribution of largest read fractions for all combinations that contain reads in multiple samples.

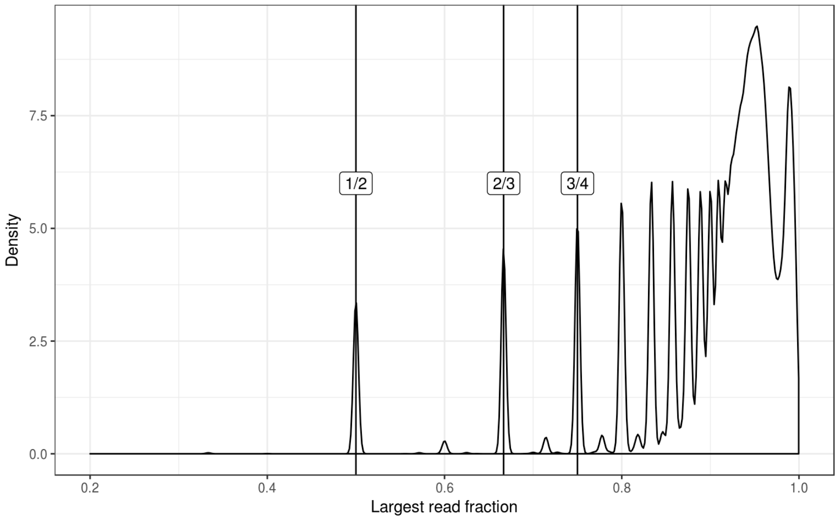

Supplementary Figure 42: Distribution of the largest read fractions for all combinations that are present in multiple samples in dataset 2, sequenced on the HiSeq 4000. Combinations where all reads existed in one sample are not considered here.

The periodic spikes in density at particular values are driven by the discreteness of count data. Three of these are annotated - a largest read fraction of 0.5 may indicate molecules with one read in each of two samples; a value of 0.66 indicates molecules with two reads in one sample, and one in

another, and so on. The greatest density is observed at values close to 1. This represents combinations where the vast majority of reads are allocated to a single sample, presumably the sample of origin.

We can visualize the high density at large values with a cumulative distribution function (Supplementary Figure 43). Over 80% of swapped molecules have a largest read fraction of above 0.8, and over 60% above 0.9.

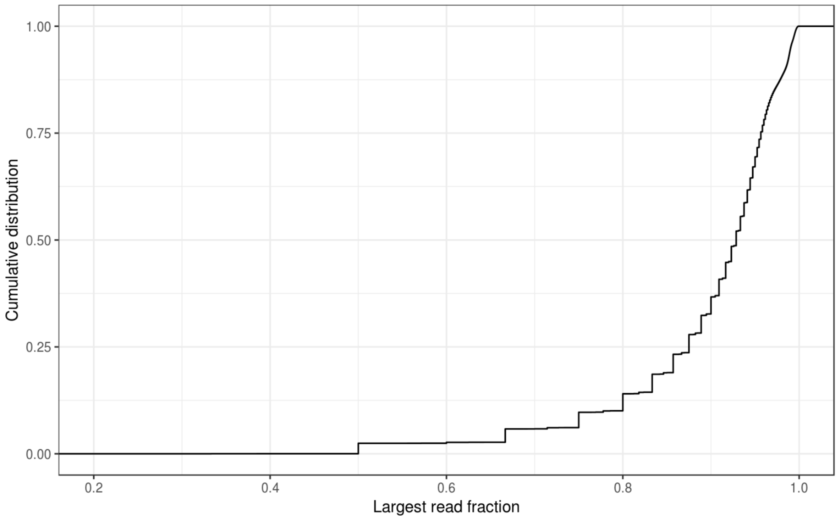

Supplementary Figure 43: Cumulative distribution of the largest read fraction for all combinations that are present in multiple samples in dataset 2, sequenced on the HiSeq 4000.

As a negative control, the same libraries were re-sequenced on the HiSeq 2500. Supplementary Table 1 presents summary values for the two sets of data. Note that many fewer molecules are swapped on the HiSeq 2500.

Supplementary Table 1: Library summary statistics. The swapped fraction is defined as the fraction of molecules (identical UMI, cell barcode, aligned gene) observed in more than one sample.

	**Reads**	**Molecules**	**Swapped fraction**
Hiseq4000	626,168,518	53,636,695	0.071033
HiSeq2500	268,768,037	44,062,616	0.000487

Supplementary Figures 44 and 45 overlay the HiSeq 2500 data over the HiSeq 4000 data.

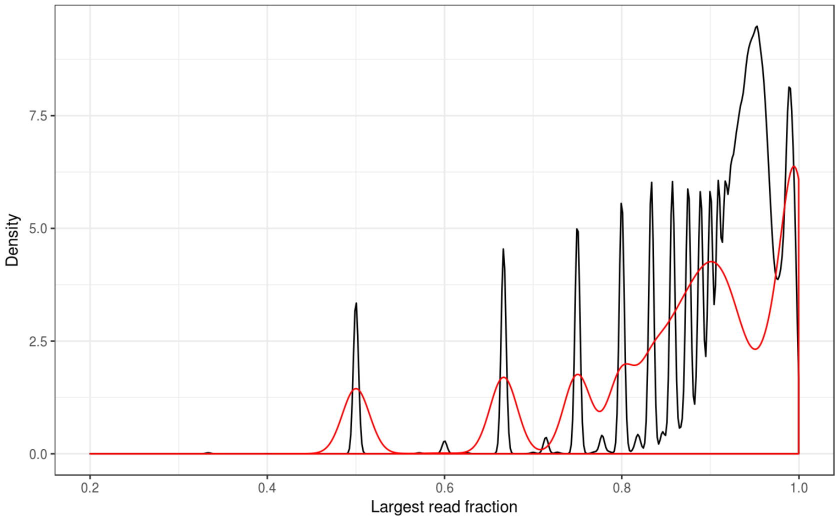

Supplementary Figure 44: Distribution of the largest read fractions for all combinations that are present in multiple samples in dataset 2, sequenced on the HiSeq 4000 (black) or HiSeq 2500 (red).

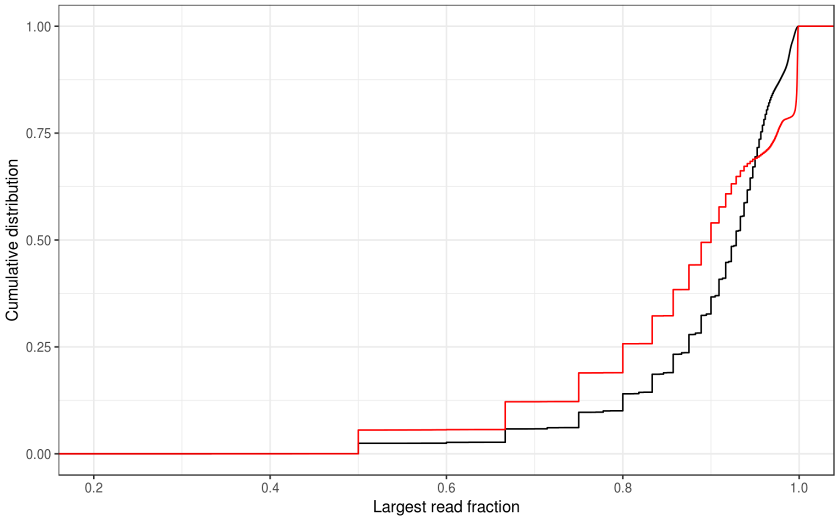

Supplementary Figure 45: Cumulative distribution of the largest read fraction for all combinations that are present in multiple samples in dataset 2, sequenced on the HiSeq 4000 (black) or HiSeq 2500 (red).

Description of the method

We have implemented an algorithm to exclude molecules that were swapped between single-cell 10X Genomics libraries in the *DropletUtils (http://bioconductor.org/packages/DropletUtils)* package. We perform the following steps:

1. Identify reads that are present in two or more different samples, yet contain the same combination of cell barcode, UMI, and aligned gene.
2. For each combination, calculate the fraction of all reads that was observed in each sample.
3. If the reads derive mostly from a single sample (largest read fraction $\geq$ 0.8), this sample is assumed to be the sample of origin, and reads in all other samples are assumed to be generated by swapping. Thus, we exclude this combination from the UMI count in all samples other than the sample of origin.
4. If the reads are relatively evenly spread across samples (largest read fraction $<$ 0.8), we cannot reliably identify the sample of origin. We therefore remove the combination from the UMI count in all samples.

Using this method, we excluded 0.715% of molecules in the HiSeq 4000 data.

Testing the method on real data

To test the effect of molecule exclusion on the data, we called cells from the processed and raw count matrices. If we successfully removed swapped reads, we should eliminate the artefactual cells that we identified in Samples B1 and B2 above (see section 4.4, **Artefactual cells due to barcode swapping in compromised samples**). We used the `emptyDrops` function from the *DropletUtils (https://github.com/MarioniLab/DropletUtils)* package to detect cells, specifiying an FDR threshold of 1% and a minimum library size of 1000 molecules. This method tests whether the expression profile for a cell barcode is significantly different from the pool of background RNA, to distinguish cell-containing and empty droplets. Importantly, this method will not

be affected by the 99th percentile effects described in Supplementary Figure 36. The results of cell calling are shown in Supplementary Table 2 and visualized in Supplementary Figure 46.

Supplementary Table 2: Number of cells called before and after swapped molecule processing.

Sample	Cells called (all molecules)	Cells called (unswapped molecules)
C1	644	638
A2	473	458
D2	161	143
A1	347	336
B2	283	13
B1	312	11
C2	1229	1212
D1	555	536

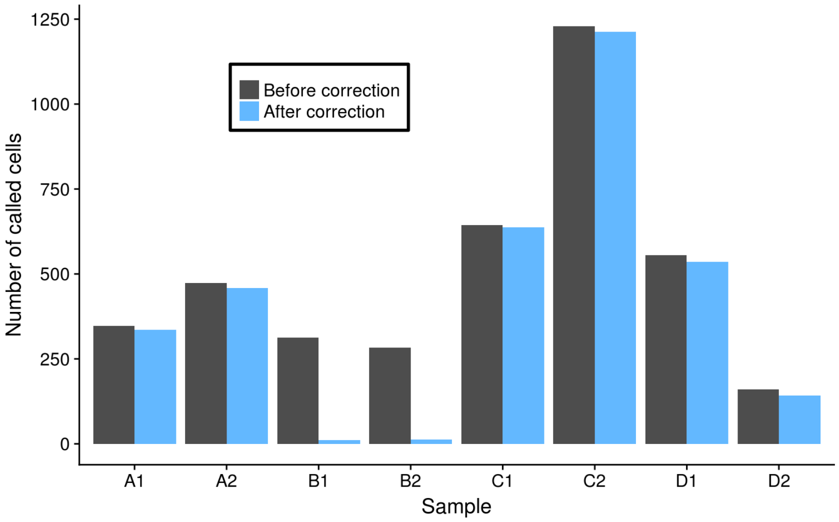

Supplementary Figure 46: Number of cells called in each sample of dataset 2 (sequenced on the HiSeq 4000) before and after swapped molecule removal. Note that the number of called cells is different to those shown earlier in this document, as this figure uses emptyDrops while the earlier figures use the CellRanger algorithm.

Our molecule exclusion method successfully removes the artefactual cells in B1 and B2. This suggests that, once swapped molecules are removed, the barcode libraries return to their background-like appearance and are correctly discarded by `emptyDrops` .

Molecule-based exclusion is preferable to cell-based exclusion, which would have resulted in the loss of all barcodes shared between B1/B2 and the other samples. However, our method still resulted in the loss of 86 cells from the other samples (2.52% of pre-correction cells). This is due to the removal of swapping contributions that inflate the total UMI count and encourage detection of potentially spurious cells. (Remember that, regardless of the number of reads, each combination will still contribute a single UMI count to a sample.)

As previously mentioned, CellRanger will always call some barcodes as cells, regardless of whether they are derived from swapping (again, as of at least version 2.1.0). As such, CellRanger fails to eliminate the artefactual cells in samples B1 and B2, even after we have removed the swapped transcripts (Supplementary Figure 47). We therefore recommend `emptyDrops` for cell calling, as it is more suitable for use with our molecule exclusion approach.

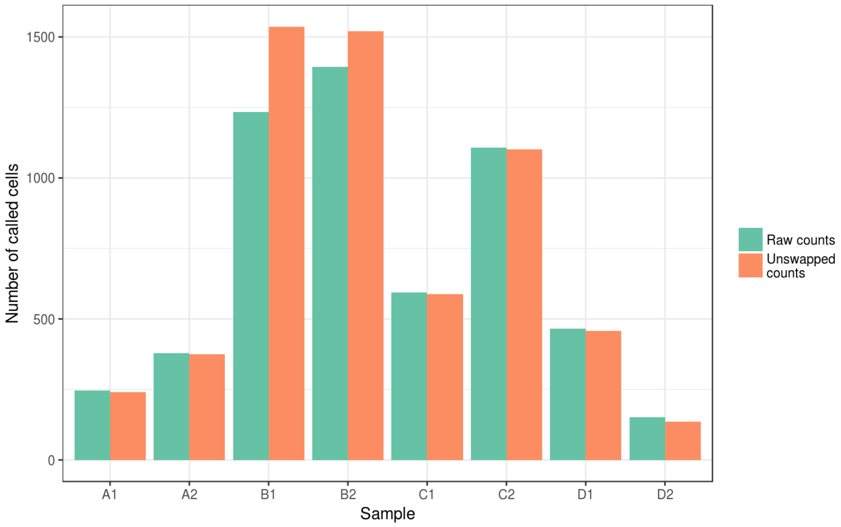

Supplementary Figure 47: Number of cells called in each sample of dataset 2 (sequenced on the HiSeq 4000) before and after swapped molecule removal, using the CellRanger algorithm for cell calling.

Testing the method on independent experiments

Finally, we applied our molecule exclusion method on two different datasets that were processed and sequenced separately. We do this as a test on the precision as the method: with two completely separate experiments there is no swapping, and therefore we should observe no or very few molecules sharing UMI, aligned gene, and cell barcode between the two datasets. We used the HiSeq 2500 dataset described previously in the section *Artefactual cells appear due to barcode swapping in compromised samples*, termed Experiment A, and a complete replicate of the same experiment that was processed and sequenced (again on the HiSeq 2500) at a later date, termed Experiment B. Dataset B is the same data that was analysed in Bach et al. (2017). A summary of the two experiments is shown in Supplementary Table 3, highlighting the number of reads generated in each experiment and the fraction of molecules within each experiment that were deemed to have swapped (according to our method).

Supplementary Table 3: Summary of the two different experiments.

Metric	Experiment.A	Experiment.B
Number of molecules	44062616	241978544
Number of swapped molecules	26105	46211
Swapped fraction	0.0592%	0.0191%

We then applied our molecule exclusion method to identity the UMI-gene-cell barcode combinations that were present in both of these two experiments, as if they were each a single sample in a multiplexed experiment. Only 688 combinations were observed in both datasets (0.000241% of all observed combinations), which is considerably fewer than the number of swapped

molecules observed within the truly multiplexed samples in each experiment (Supplementary Table 3). This demonstrates the specificity of our molecule exclusion method for removing swapping artefacts in 10X data.

Using this framework, we can estimate a swapping fraction for this 10X data by considering the fraction of all reads that we deem to have swapped. Our swap-identification method provides a swapping fraction of 0.978% on the HiSeq 4000, and 0.0139% on the HiSeq 2500. This is comparable to the swapping fraction we estimated for Smart-seq2 data, with a modest discrepency attributable to differences between the assays (Costello et al. 2017). Consistent with our previous results, we observe an order of magnitude difference between swapping fractions on each technology.

Note that this swapped fraction is harder to interpret than the fractions for plate based data for a number of reasons:

- As previously mentioned, a swapped read does not necessarily find itself in the library of another cell. Instead, it may result in the generation of a new artefactual cell library. This complicates interpretation of the effects of a given swapping fraction.
- The 10X swapped fraction estimate relies on our sample-of-origin identification procedure (i.e. fraction of reads $\geq$ 80%). Molecules without a clear sample-of-origin are all considered to be swapped, which yields inaccurate estimates of the swapped fraction.
- Each 10X sample is actually labelled by a mixture of four sample barcodes. We have only considered swapping between samples, not the swapping of individual barcodes. This is therefore not an estimate of the molecular swapping rate

Supplementary Note 7: Experimental solutions for barcode swapping

One proposed solution for the swapping problem are unique-at-both-ends indices. In these experiments, a cDNA molecule in a given cell's library is indexed with a unique barcode at each end. These barcodes are never reused for any other cell library in the same multiplexed set. A single barcode swap therefore moves a sequencing read into an unused barcode combination, not into another cell library. This is an effective solution for the multiplexing of a relatively low number of libraries for sequencing (e.g., Nugen provide sets of 96 pairs of indexes (https://www.nugen.com/content/nugen-introduces-unique-indexing-solutions-illumina's-high-capacity-sequencing-platforms)).

However, for single-cell RNA-seq, it may be desirable to sequence many hundreds of multiplexed samples (i.e., cells) together, particularly as sequencing facilities transition towards the use of higher-throughput machines. Use of unique-at-both-ends indices may not be feasible in these experiments, because of the loss of combinatorial complexity provided by the reuse of barcodes in a multiplexed set of libraries. Consider the situation where we have ζ unique barcode sequences. If barcodes are reused between cells in unique combinations, the maximum number of samples that can be multiplexed together is:

$$\left(\frac{\zeta}{2}\right)^2,$$

assuming that $\frac{\zeta}{2}$ barcodes are exclusively used for 5' or 3' indexing. In contrast, the maximum number of combinations for unique-at-both-ends indexing is:

$$\frac{\zeta}{2}.$$

Clearly, unique-at-both-ends indexing severely restricts the throughput of multiplexing strategies.

In practice, barcodes are often used in a 96-well plate (of dimension 12 x 8). For every additional 20 barcodes that are available, assume that 8 are used to index rows (say, 5' indexing), and 12 are used to index columns (3' indexing). Here, the number of possible barcode combinations is:

$$\frac{8\zeta}{20} \times \frac{12\zeta}{20} = \frac{6\zeta^2}{25}$$

Again, this approach allows the multiplexing of very many more samples than unique-at-both-ends approaches. The difference in scaling of these values is illustrated in Supplementary Figure 48.

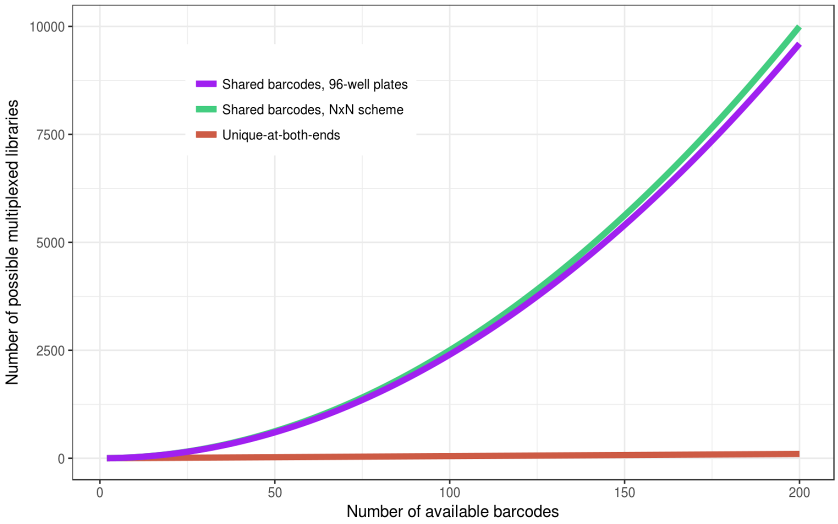

Supplementary Figure 48: Maximum number of libraries that can be multiplexed under different labelling schemes, as a function of the number of available barcodes.

Use of unique-at-both-ends barcoding is particularly problematic for methods such as sci-Seq (Cao et al. 2017), where the reuse of barcodes between cells generates the combinatorial complexity that allows massively high-throughput generation of cell libraries. Additionally, the use of droplet-based protocols (e.g. 10X Genomics) is increasingly popular. In these experiments, samples are labelled with a single index, and so are not compatible with the unique-at-both-ends indexing approach.

While we would encourage the use of experimental designs with unique-at-both-ends indexing where possible, it is clearly not a suitable approach for all experiments. This motivates our development of computational methods to address barcode swapping, particularly for droplet-based scRNA-seq data.

Supplementary Note 8: Manuscript figures

Larger versions of the figures used in the manuscript that were generated using R are present below.

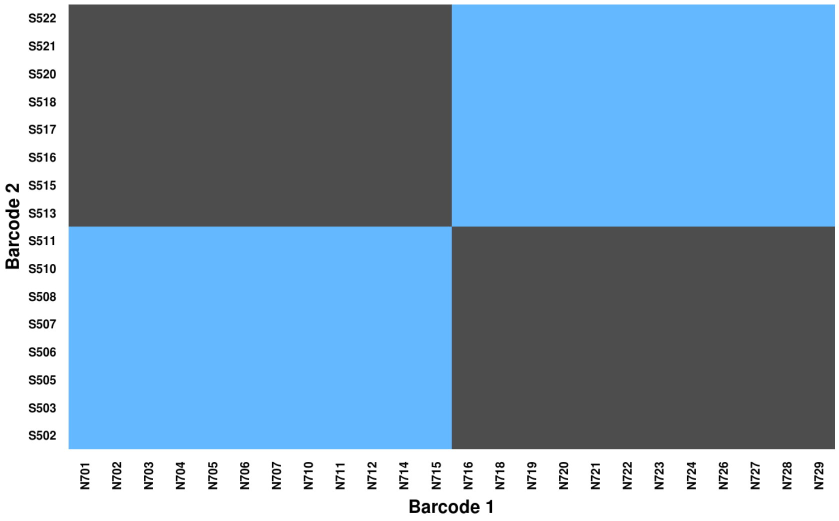

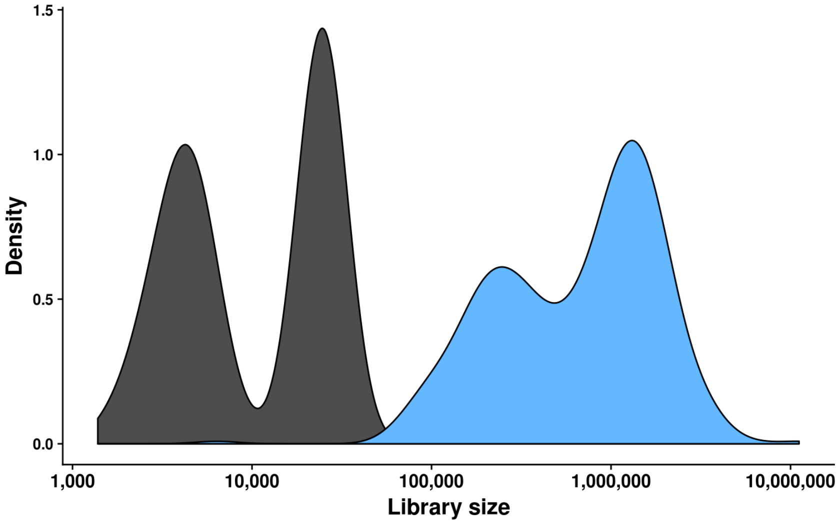

Supplementary Figure 2b

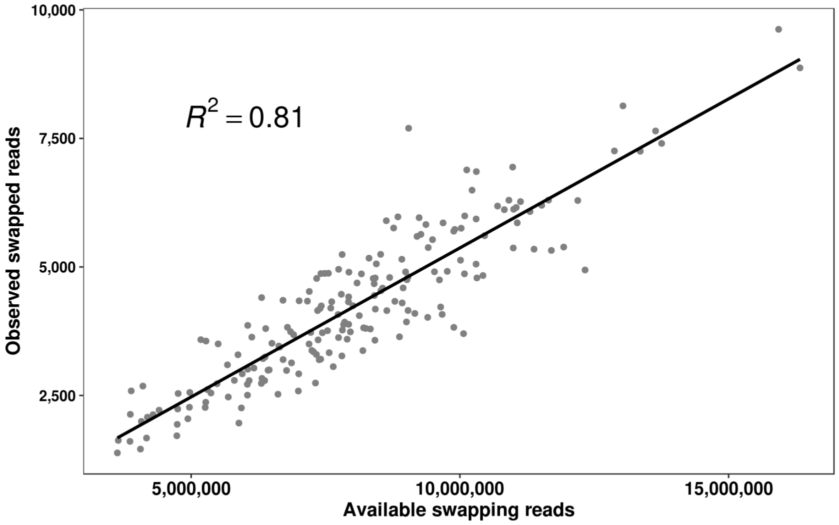

Supplementary Figure 2c

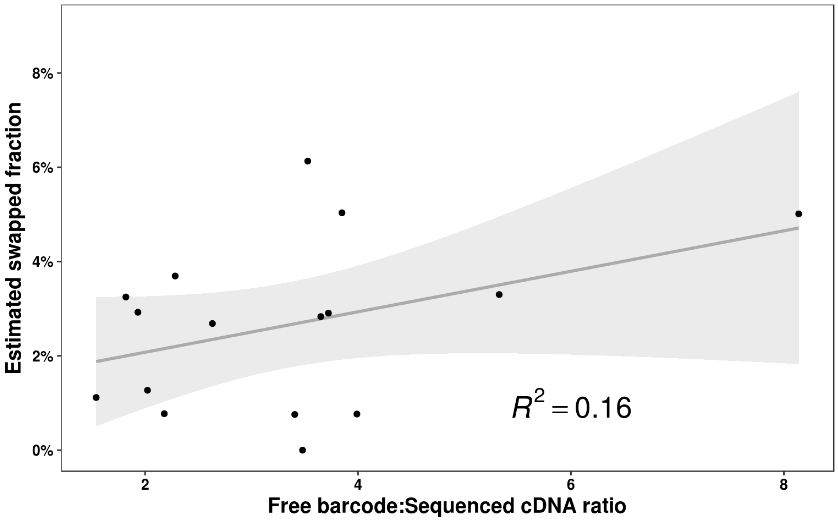

Supplementary Figure 2

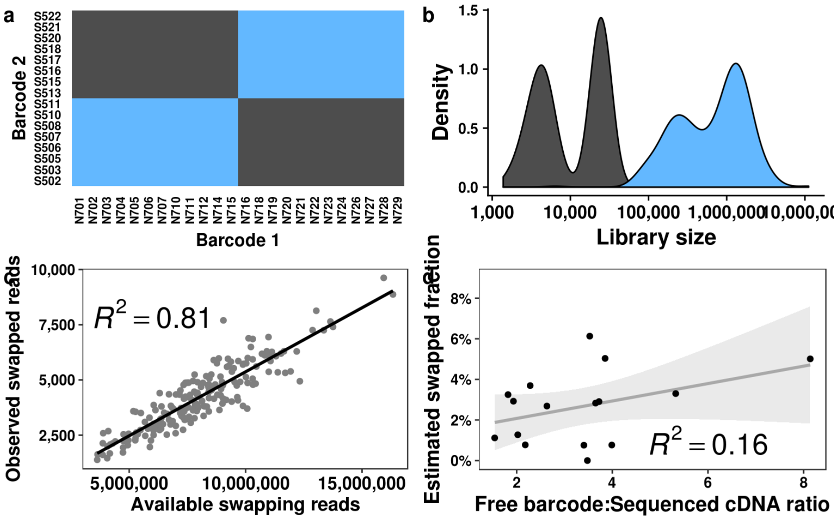

Supplementary Figure 3a

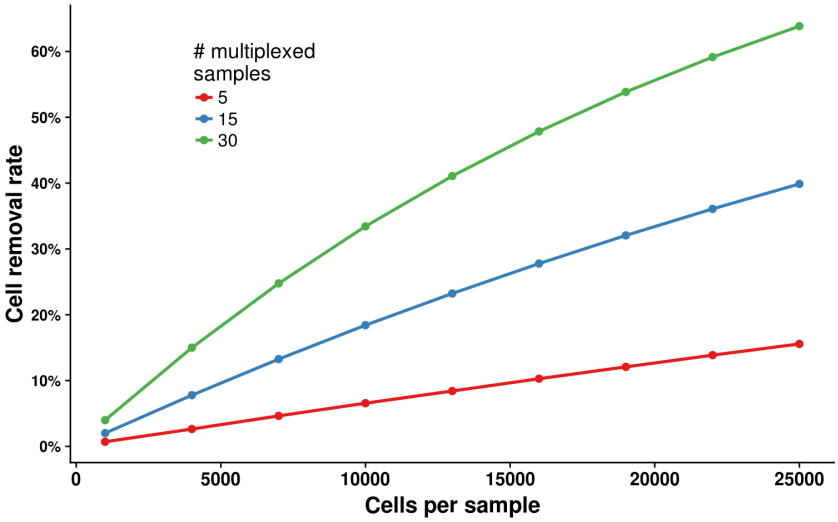

Supplementary Figure 3c

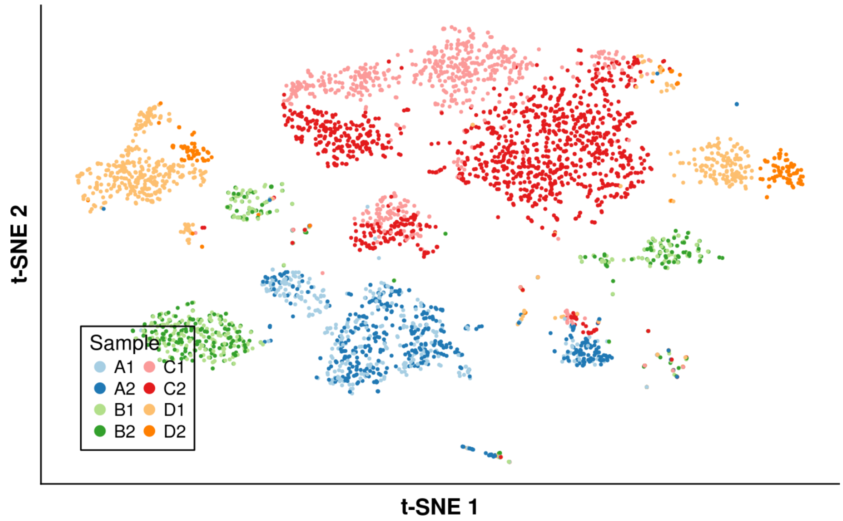

Supplementary Figure 3d

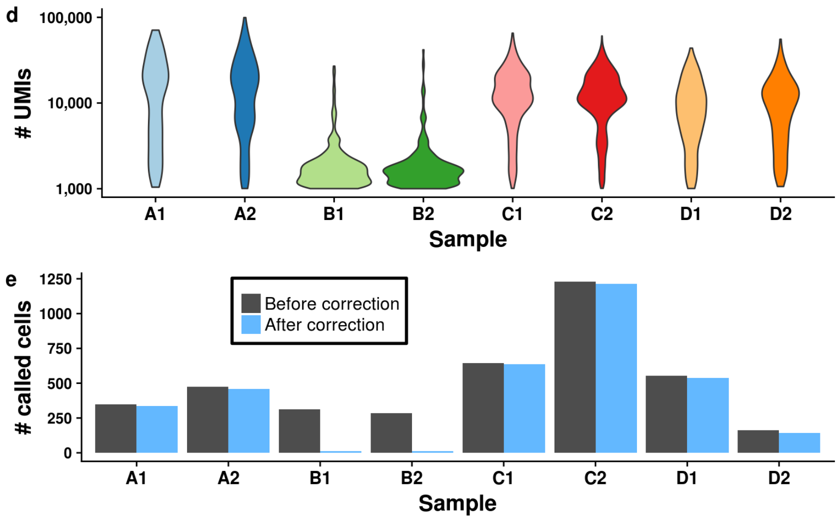

Supplementary Figure 3

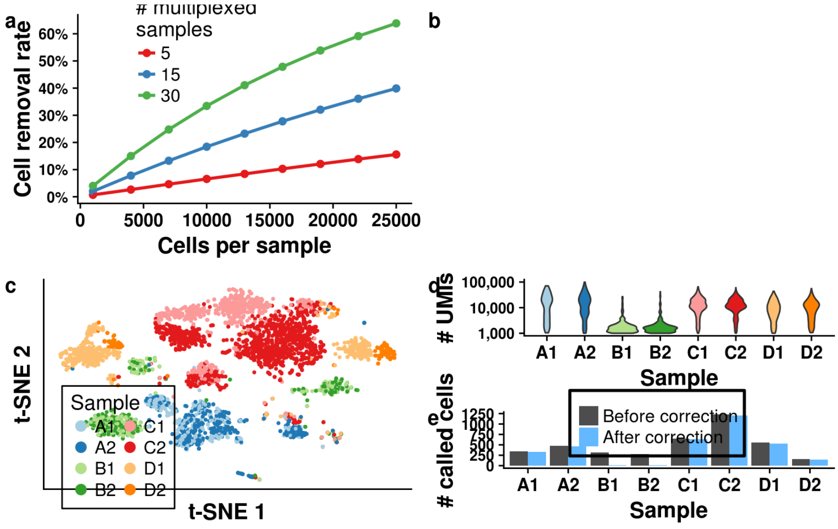

References

Bach, Karsten, Sara Pensa, Marta Grzelak, James Hadfield, David J. Adams, John C. Marioni, and Walid T. Khaled. 2017. "Differentiation Dynamics of Mammary Epithelial Cells Revealed by Single-Cell RNA Sequencing." *Nature Communications* 8 (1): 2128. doi:10.1038/s41467-017-02001-5 (https://doi.org/10.1038/s41467-017-02001-5).

Cao, Junyue, Jonathan S. Packer, Vijay Ramani, Darren A. Cusanovich, Chau Huynh, Riza Daza, Xiaojie Qiu, et al. 2017. "Comprehensive Single-Cell Transcriptional Profiling of a Multicellular Organism." *Science (New York, N.Y.)* 357 (6352): 661–67. doi:10.1126/science.aam8940 (https://doi.org/10.1126/science.aam8940).

Costello, Maura, Mark Fleharty, Justin Abreu, Yossi Farjoun, Steven Ferriera, Laurie Holmes, Tom Howd, et al. 2017. "Characterization and Remediation of Sample Index Swaps by Non-Redundant Dual Indexing on Massively Parallel Sequencing Platforms." *BioRxiv*, October, 200790. doi:10.1101/200790 (https://doi.org/10.1101/200790).

Illumina. 2017. "Effects of Index Misassignment on Multiplexing and Downstream Analysis." [White Paper]. Accessed June 21. https://www.illumina.com/content/dam/illumina-marketing/documents/products/whitepapers/index-hopping-white-paper-770-2017-004.pdf?linkId=36607862 (https://www.illumina.com/content/dam/illumina-marketing/documents/products/whitepapers/index-hopping-white-paper-770-2017-004.pdf?linkId=36607862).

Liao, Yang, Gordon K. Smyth, and Wei Shi. 2013. "The Subread Aligner: Fast, Accurate and Scalable Read Mapping by Seed-and-Vote." *Nucleic Acids Research* 41 (10): e108. doi:10.1093/nar/gkt214 (https://doi.org/10.1093/nar/gkt214).

———. 2014. "featureCounts: An Efficient General Purpose Program for Assigning Sequence Reads to Genomic Features." *Bioinformatics (Oxford, England)* 30 (7): 923–30. doi:10.1093/bioinformatics/btt656 (https://doi.org/10.1093/bioinformatics/btt656).

Marioni, John C., Christopher E. Mason, Shrikant M. Mane, Matthew Stephens, and Yoav Gilad. 2008. "RNA-Seq: An Assessment of Technical Reproducibility and Comparison with Gene Expression Arrays." *Genome Research* 18 (9): 1509–17. doi:10.1101/gr.079558.108 (https://doi.org/10.1101/gr.079558.108).

Nestorowa, Sonia, Fiona K. Hamey, Blanca Pijuan Sala, Evangelia Diamanti, Mairi Shepherd, Elisa Laurenti, Nicola K. Wilson, David G. Kent, and Berthold Göttgens. 2016. "A Single Cell Resolution Map of Mouse Haematopoietic Stem and Progenitor Cell Differentiation." *Blood*, January, blood–2016–05–716480. doi:10.1182/blood-2016-05-716480 (https://doi.org/10.1182/blood-2016-05-716480).

Picelli, Simone, Omid R. Faridani, Asa K. Bjorklund, Gosta Winberg, Sven Sagasser, and Rickard Sandberg. 2014. "Full-Length RNA-Seq from Single Cells Using Smart-Seq2." *Nature Protocols* 9 (1): 171–81. doi:10.1038/nprot.2014.006 (https://doi.org/10.1038/nprot.2014.006).

Richard, Arianne C, Aaron TL Lun, W Lau, B Göttgens, JC Marioni, and GM Griffiths. 2018. "T Cell Cytolytic Capacity Is Independent of Stimulation Strength." *Nature Immunology* In press.

Sinha, Rahul, Geoff Stanley, Gunsagar Singh Gulati, Camille Ezran, Kyle Joseph Travaglini, Eric Wei, Charles Kwok Fai Chan, et al. 2017. "Index Switching Causes 'Spreading-Of-Signal' Among Multiplexed Samples In Illumina HiSeq 4000 DNA Sequencing." *BioRxiv*, April. doi:10.1101/125724 (https://doi.org/10.1101/125724).

Xu, Chen, and Zhengchang Su. 2015. “Identification of Cell Types from Single-Cell Transcriptomes Using a Novel Clustering Method.” *Bioinformatics* 31 (12): 1974–80. doi:10.1093/bioinformatics/btv088 (https://doi.org/10.1093/bioinformatics/btv088).

Zheng, Grace X. Y., Jessica M. Terry, Phillip Belgrader, Paul Ryvkin, Zachary W. Bent, Ryan Wilson, Solongo B. Ziraldo, et al. 2017. “Massively Parallel Digital Transcriptional Profiling of Single Cells.” *Nature Communications* 8 (January): 14049. http://dx.doi.org/10.1038/ncomms14049 (http://dx.doi.org/10.1038/ncomms14049).
